# Integrating AI in Medicinal Chemistry for Accelerated Drug Discovery: A Comprehensive SAR (CSAR) Optimization Strategy and Discovery of Potent ALDH3A1 Inhibitors

**DOI:** 10.1021/acs.jmedchem.6c00537

**Published:** 2026-05-30

**Authors:** Sankalp Jain, Adam Yasgar, Anu Dalal, Aleksandra Nilova, Marissa Davies, Bolormaa Baljinnyam, Yanyan Qu, John-Paul Denson, Dominic Esposito, Dingyin Tao, Shyh-Ming Yang, Daniel C. Talley, Anton Simeonov, Natalia J. Martinez, Ganesha Rai, Alexey V. Zakharov

**Affiliations:** † National Center for Advancing Translational Sciences (NCATS), 2511 National Institutes of Health, 9800 Medical Center Drive, Rockville, Maryland 20850, United States; ‡ Protein Expression Laboratory, Cancer Research Technology Program, Frederick National Laboratory for Cancer Research, Frederick, Maryland 21701, United States

## Abstract

Developing potent, selective small-molecule inhibitors remains a major challenge in drug discovery. ALDH3A1, a detoxifying aldehyde dehydrogenase isoform implicated in cancer and neurodegeneration, is a promising yet underexplored therapeutic target. To accelerate inhibitor optimization, we developed an AI-guided, reaction-based hit-to-lead workflow combining sequential reaction enumeration, pharmacophore-informed docking, and predictive modeling to support scalable SAR expansion. Applied to ALDH3A1, two rounds of enumeration using Enamine building blocks generated about 250,000 virtual analogues. Combined deep learning and docking-based triage prioritized 150 compounds for synthesis, leading to a roughly 1,000-fold improvement in biochemical potency from 1.41 μM to 1 nM for NCATS-SM0707, together with 4 nM cellular activity for NCATS-SM0708. Crucially, this methodology can be expanded by applying various chemical reactions at different positions. This study highlights CSAR as a scalable, generalizable complementary strategy to accelerate hit optimization through reaction-based enumeration and AI-guided prioritization of larger synthetically accessible chemical space.

## Graphical abstract

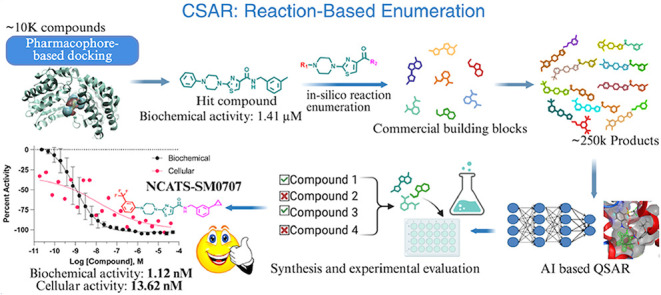

## Introduction

The discovery and optimization of small molecules with therapeutic potential remains a central challenge in drug discovery and typically requires multiple design–make–test cycles. Although advances in high-throughput screening (HTS), structure-based modeling, and machine learning have improved early hit identification, the subsequent progression from initial hits to selective, potent, and pharmacologically viable lead compounds still requires substantial time and resources, especially for targets with limited chemical tractability.^1–4^ Traditional drug discovery programs fall short due to insufficient structure–activity relationship (SAR) exploration limited to a finite number of building blocks and restricted access to chemically diverse yet synthetically feasible analogs. As therapeutic targets grow increasingly complex and selective, there is a pressing need for scalable strategies that can systematically explore SAR utilizing larger chemical space while accounting for the practicality of synthesis, biological activity, drug-like properties^5,6^ and improving intellectual property.

Aldehyde dehydrogenase 3A1 (ALDH3A1) is one such pharmacologically relevant target. A member of the NAD­(P)+-dependent ALDH enzyme family, ALDH3A1 plays a role in detoxifying lipid-derived aldehydes and has been implicated in redox regulation, chemoresistance, and immune evasion.^7,8^ Overexpressed in solid tumors such as lung, breast, and head and neck cancers, ALDH3A1 has been linked to tumor progression and poor clinical outcomes.^9^ With its growing biological relevance over the last two decades, multiple inhibitors have been disclosed,^10^ including MI 192 (NCGC00480746), recently identified by our center.^11^ Our experience with ALDH3A1 makes it an ideal candidate to test comprehensive structure–activity relationship (CSAR) strategy.

Traditional medicinal chemistry approaches, such as stepwise R-group optimization and Topliss-guided substitution, remain foundational to lead optimization.^12,13^ These involve iterative synthesis and testing of analogs guided by SAR insights and synthetic tractability using a finite chemical space. In a classic Topliss strategy, substitutions are systematically explored in a sequential manner, starting with an R_1_ position and progressing to adjacent sites such as R_2_. Analog-by-catalog strategies are further constrained by commercial availability, frequently restricting SAR development to familiar chemical space.^14,15^ Although powerful and logic-driven, these methods are inherently limited in scale, slow process, and are often unable to fully access the breadth of large chemical space necessary to uncover high-quality, novel, and selective chemical matters.

To overcome these constraints, computational methods such as reaction-based enumeration and machine learning offer practical, scalable solutions.^16–18^ These tools enable the generation of synthetically accessible analogs based on known scaffolds using curated building blocks and validated reaction templates. Critically, they allow access to vast regions of chemical space that are otherwise infeasible to explore manually and help identify nonobvious SAR trends through data-driven triaging.^19^ When combined with docking and predictive models trained on experimental bioactivity data, these strategies support hypothesis-driven design at scale, reduce selection bias, and promote a more practical and efficient SAR workflow.

Several platforms such as REINVENT,^20^ MegaSyn,^21^ and AutoSynRoute^22^ exemplify advances in generative design and synthesis planning. REINVENT applies reinforcement learning to guide analog generation toward desired activity profiles.^20^ MegaSyn incorporates synthetic feasibility scoring to suggest viable compounds.^21^ AutoSynRoute supports retrosynthetic planning and prioritization.^22^ While powerful for scaffold hopping and idea generation, these platforms are typically designed for early stage exploration and are not optimized for scaffold-specific SAR expansion. Their generalized reaction rules often lack the detailed chemical context needed to fine-tune scaffold modifications for targets like ALDH3A1, where even small changes in substituents lead to dramatic shifts in activity or isoform selectivity.^23,24^

To bridge this gap, we developed a reaction-based enumeration strategy focused on scaffold-anchored SAR development (Figure 1). Our approach mirrors classical substitution logic (e.g., R_1_ → R_2_) but is amplified by in silico scalability through our reaction templates. Starting with over one million building blocks from Enamine and two tractable reaction templates to modify around the two regions of the hit scaffold, we constructed a virtual library of ∼250,000 synthesizable analogs around the hit. Beyond the demonstrated two-round campaign, this modular framework is inherently extensible, allowing for the integration of diverse chemical transformations at different positions of the molecule to comprehensively explore and optimize lead scaffolds. This library was refined through pharmacophore-based docking analysis of ALDH3A1 binding interactions, and deep learning consensus architecture (DLCA) model trained on experimental biochemical data. The resulting prioritization enabled rapid selection of analogs with optimal tractability, predicted potency, and SAR. Following two rounds of enumeration and synthesis, generating 72 analogs in the first iteration and 78 in the second, this process led to the identification of a lead compound with 1 nM biochemical potency, 14 nM cellular activity, and marked ALDH isoform selectivity, together with initial in vitro ADME characterization.^25,26^ In the present study, this workflow was demonstrated through reaction-defined exploration of two medicinal chemistry vectors. More broadly, however, the underlying framework is not limited to the transformations demonstrated here and can be extended through additional reaction schemes, such as linker modifications and scaffold diversification, combined with iterative design cycles when broader medicinal chemistry exploration is required. Our results further demonstrate that this generative workflow can accelerate the development of potent inhibitors by combining the insights of classical medicinal chemistry with scalable in-silico methods to rapidly generate lead compounds, even for targets lacking established tool compounds or well-defined SAR.

**Figure 1:**
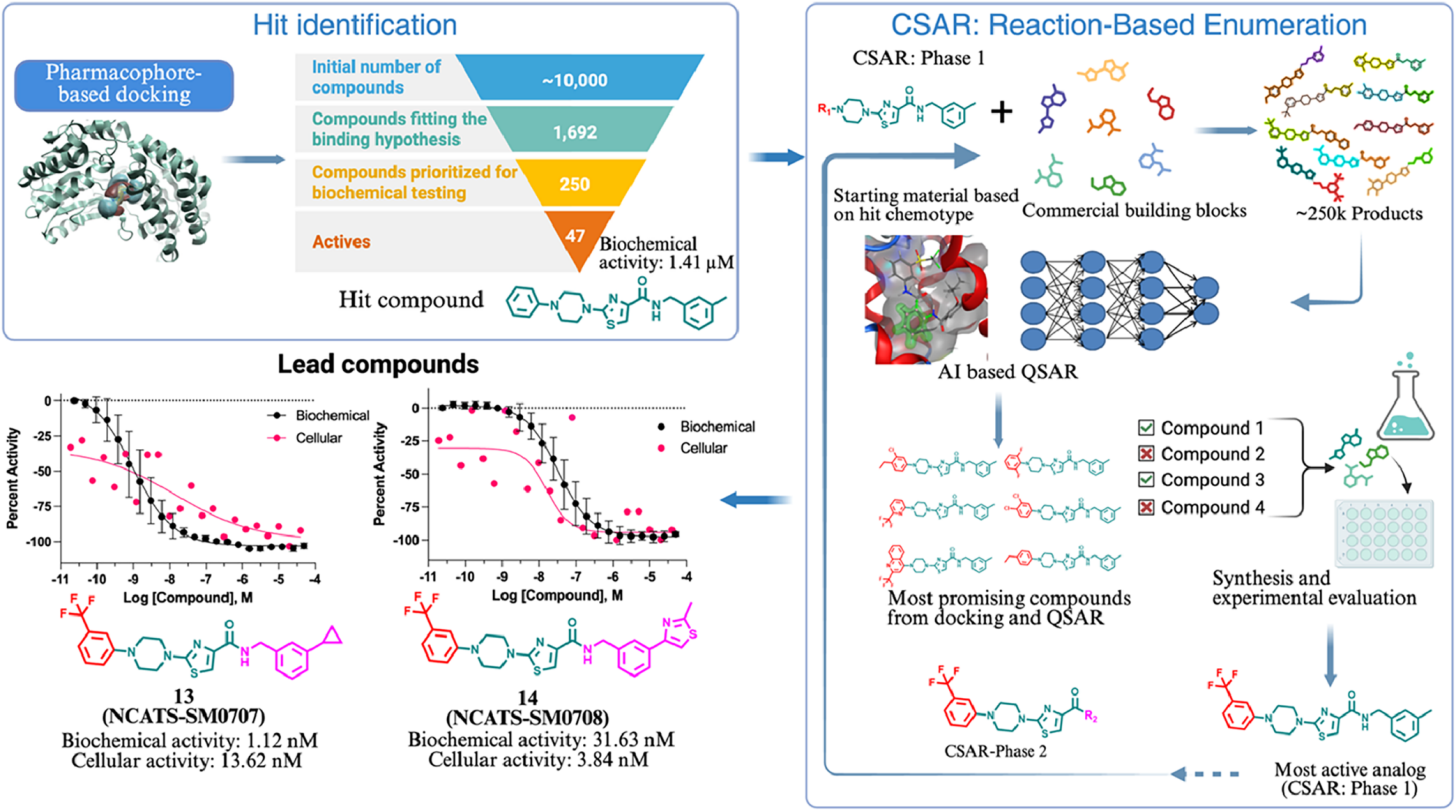
Study workflow. Flowchart summarizing hit identification by pharmacophore-based docking, reaction-based enumeration across two design-make-test iterations, QSAR modeling for prioritization, and biochemical and cellular validation. Two rounds of enumeration and synthesis generated 72 analogs (Round 1) and 78 analogs (Round 2), leading to a lead compound (NCATS-SM0707) with 1 nM biochemical potency and ∼14 nM cellular activity and another (NCATS-SM0708) with ∼32 nM biochemical potency and ∼ 4 nM cellular activity.

## Results

### Hit Identification through Pharmacophore-Based Virtual Screening

We employed a structure-guided virtual screening strategy to identify hits suitable for systematic SAR expansion using ALDH3A1 as our model protein. A pharmacophore model was constructed from the ALDH3A1 crystal structure (PDB ID: 4H80), capturing key interaction features within the substrate-binding tunnel, including hydrogen bond acceptors, hydrophobic elements, and an aromatic core positioned near the cofactor-binding site. This model was applied in combination with molecular docking to a curated library of ∼10,000 chemically diverse compounds from the NCATS internal screening collection, resulting in 1,692 candidates with compatible binding modes. From these, 250 compounds were prioritized for testing in the ALDH3A1 biochemical assay based on pharmacophore alignment, docking score, and structural diversity. Briefly, the biochemical assay utilizes the substrate benzaldehyde and the cofactor NAD­(P)+ coupled to a diaphorase and resazurin readout and it is formatted to 1,536-well plates (see Experimental Section). After testing compounds in dose–response format, 47 out 250 showed activity, which led to 18.8% hit rate. From 47 compounds 34 compounds exhibited IC_50_ values below 30 μM corresponding to a hit rate of 13.6% (Supporting Information Table S1; Figure 2). To confirm that the activity extended beyond the original hits, we searched the NCATS internal collection (∼150,000 compounds) for close analogs of the most potent compounds, as such related molecules would typically be expected to show comparable activity. A total of 232 analogs were identified and tested in the same biochemical assay, resulting in 65 additional actives (IC_50_ < 30 μM), corresponding to a hit rate of 28% (Supporting Information Table S2).

**Figure 2:**
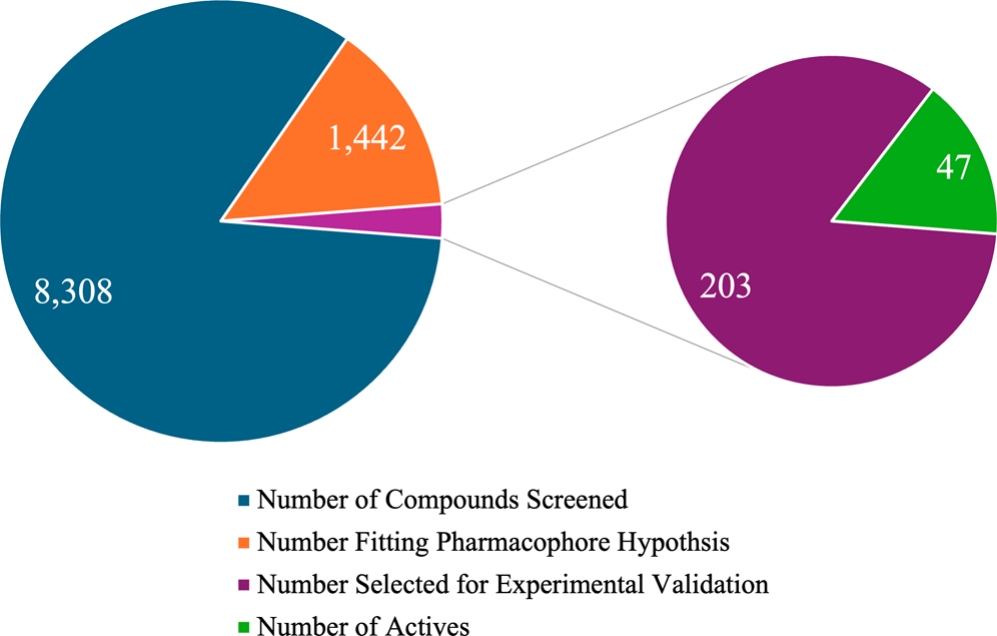
Docking triage and experimental outcomes. Pharmacophore-based docking was applied to a curated ∼10,000-compound NCATS library, resulting in 1,692 compounds consistent with the pharmacophore hypothesis. From these, 250 compounds were prioritized for biochemical testing based on pharmacophore alignment, docking score, and structural diversity, resulting in 47 actives.

The combined set of active compounds resulted in five distinct hits. These hits exhibited IC_50_ values predominantly in the 5–20 μM range. While none achieved high potency at this stage, some scaffolds displayed well-defined SAR trends, with two chemotypes amenable to substitution without disrupting key binding interactions (Figure 3).

**Figure 3:**
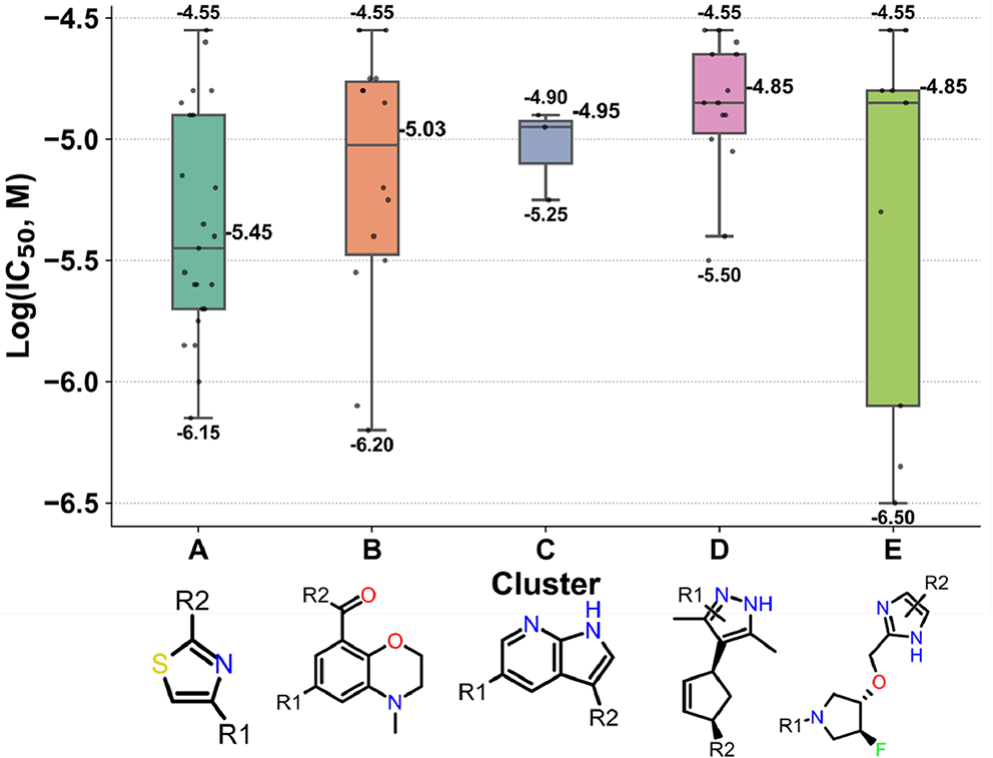
Potency distribution across five chemotypes used to guide scaffold selection. Boxplot shows log­(IC_50_) values for five representative scaffolds identified from the active compound set (IC_50_ < 30 μM). Each scaffold is shown below with substituent positions (R_1_, R_2_) indicated.

### Scaffold Selection for SAR Expansion

Among the five chemotypes identified from the combined screening and analog profiling, the thiazole-containing scaffold **A** (Figure 3) was selected for systematic SAR expansion. This scaffold was chosen based on its consistent midmicromolar potency (Table 1), early ADME profile, clear pharmacophore alignment, and well-defined functional handles suitable for further SAR modification. From a medicinal chemistry perspective, its planar aromatic core and balanced physicochemical properties provided a tractable starting point for analog design and optimization.

**Table 1:**
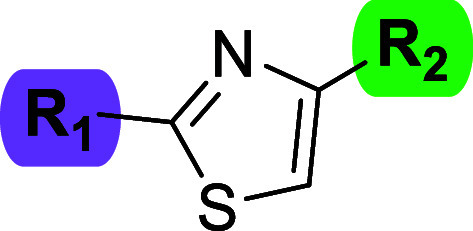

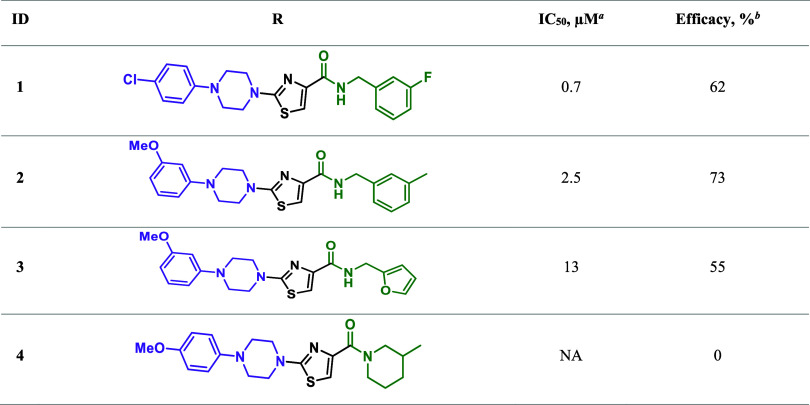
Select Example of In-House Analogs of Scaffold **A**

a IC_50_: half-maximal inhibitory concentration values obtained in 11-point dose response, measured in triplicate.

b Efficacy: maximum inhibitory effect observed in corresponding assay.

Additionally, we identified 48 analogs of **A** in the library, of which 43 compounds shared common structural features including an *N*-substituted piperazine moiety and amide functionality at the R_1_ and R_2_ positions. This group included all 23 analogs that demonstrated measurable ALDH3A1 inhibition, with IC_50_ values ranging from 0.7 μM to 28 μM and efficacy spanning 33% to 92%. Early SAR analysis around the amide substitution R_2_ revealed that various substituted benzylamines are highly preferred over heteroarylamine analogs and aliphatic derivatives. Incorporation of secondary over primary amides showed reduced activity. The scope of available in-house analogs with the variation at R_1_ position was highly limited to monosubstituted *N*-phenyl piperazines and did not lead to noticeable SAR trends. Taken together, these features positioned the scaffold as a strong candidate for reaction-based enumeration and lead development.

### CSAR Phase I: Reaction-Based Enumeration at R_1_

The first round of SAR focused on the R_1_ position of the thiazole-containing scaffold **A**, corresponding to the *N*-substituted piperazine moiety highlighted in purple in Table 1. The piperazine moiety and its substitution pattern were retained based on docking poses and QSAR predictions, which indicated that piperazine provided the most favorable scores compared with other potential replacements. From a design standpoint, R_1_ position served as a synthetically accessible handle that allowed for exploration of chemical space without replacing the core scaffold or key pharmacophoric contacts.

To access a broad and synthetically tractable chemical space around the *N*-substituted piperazine moiety, we employed an in-silico reaction-based enumeration strategy. Specifically, nucleophilic aromatic substitution of a 2-chlorothiazole intermediate (Scheme 1) was performed virtually using over one million commercially available *N*-substituted piperazine building blocks from the Enamine database.^27^ The enumeration generated 9,336 analogs with variation restricted to substituent on N atom of piperazine. Compound prioritization was guided by QSAR-based activity prediction trained on primary screen data, followed by pharmacophore-based docking (MOE) to confirm with key binding features.

**Scheme 1:**
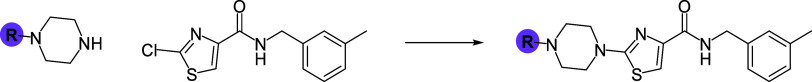
Nucleophilic Aromatic Substitution of a 2-Chlorothiazole Intermediate with *N*-Substituted Piperazines^a^

a Reaction conditions used for synthesis: DMSO, 110 °C, 6 h.

### QSAR Model Performance and Compound Selection

To prioritize compounds for synthesis, we developed a QSAR regression model based on biochemical data from the initial ALDH3A1 inhibitor screen. The model was constructed using Deep Learning Consensus Architecture (DLCA), which previously showed comparable or better performance compared to modern state of the art deep learning methods.^28,29^ It achieved an *R* ^2^ of 0.51 for 5-fold external cross validation procedure, providing sufficient predictive power to guide early phase compound selection. For comparison, alternative Random Forest models built on RDKit descriptors, Morgan fingerprints, Avalon fingerprints, AtomPair fingerprints, and their combined feature set showed lower average performance over 5-fold cross-validation than the DLCA model (Supporting Information Table S3). The 9,336 analogs generated through reaction-based enumeration were scored using this model and further evaluated using pharmacophore-based docking to assess compatibility with the ALDH3A1 active site and retention of key interaction features. Based on QSAR regression model score, docking score, and binding pose quality, 500 compounds were shortlisted for final review. This list was then jointly assessed with medicinal chemists to ensure synthetic feasibility and SAR relevance. From this refined subset, 80 building blocks were selected, sourced commercially for incorporation at the R_1_ position using the nucleophilic aromatic substitution reaction.

### Synthesis and Bioactivity of R_1_ Compounds

Of the 80 *N*-substituted piperazine building blocks selected for synthesis, 72 analogs were successfully synthesized using nucleophilic aromatic substitution reaction (Scheme 1), resulting in an overall synthetic success rate of approximately 90%. After assessing their ALDH3A1 inhibitory activity, 70 compounds (97%) demonstrated IC_50_ values below 30 μM and efficacy >30% (Supporting Information Table S4). Potency values ranged broadly, with the majority falling between 0.23–20 μM (Supporting Information Table S4). Notably, 48 analogs demonstrated improved potency relative to the original hit (IC_50_ = 1.41 μM, Efficacy = 74%), underscoring the tractability of the scaffold for SAR optimization (Figure 4 (A)). The most active analogs contain differently substituted phenyl piperazinyl groups with preference for smaller substituents on the phenyl ring (Table 2). However, substituted pyridyl piperazinyl functionalities also demonstrate good activity in the biochemical assay (compound **11**). While phenyl containing analogs have extremely low solubility, incorporation of a pyridyl moiety led to increased solubility without compromising in potency.

**Figure 4:**
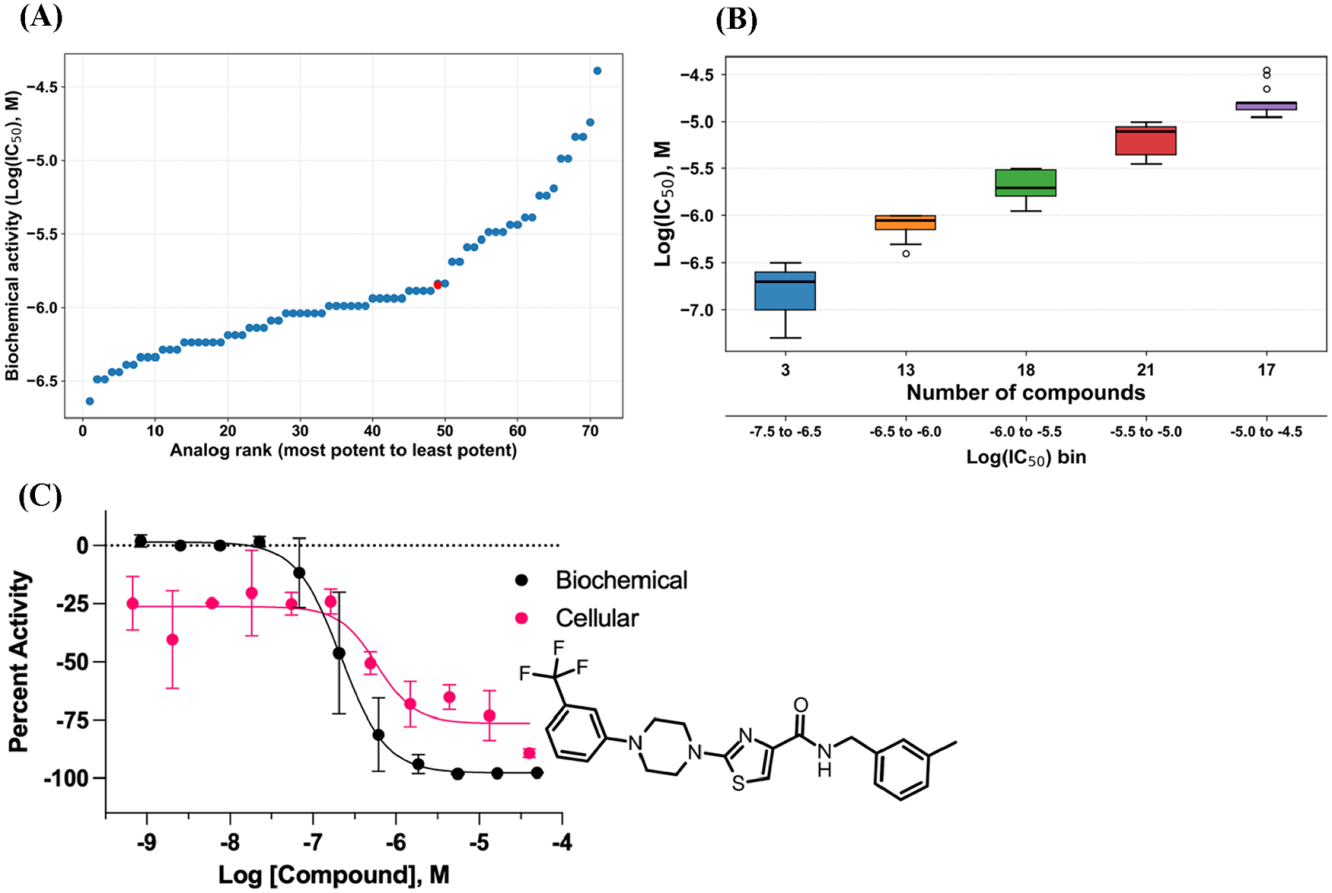
Biochemical and cellular activity of analogs synthesized from R_1_enumeration. (A) ALDH3A1 biochemical activity (Log IC_50_, μM) of 72 synthesized analogs, ranked by potency. The original hit from pharmacophore-guided screening is marked in red. A significant number of analogs showed improved activity over the initial lead. (B) Cell-based activity (Log IC_50_, μM) binned by log­(IC_50_) ranges. Multiple compounds demonstrated submicromolar potency, with cluster-specific differences in cellular response. (C) Representative dose–response curves for compound **5** (black, ALDH3A1 biochemical IC_50_ = 230 nM) and cellular potency in OE19 cells (pink, IC_50_ = 3.15 μM), confirming translation of biochemical activity to the cellular context.

**Table 2:**
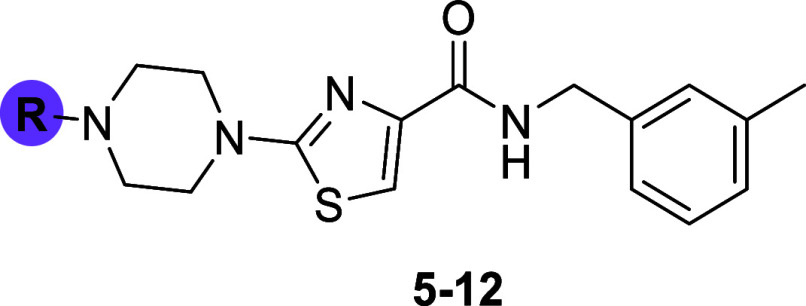

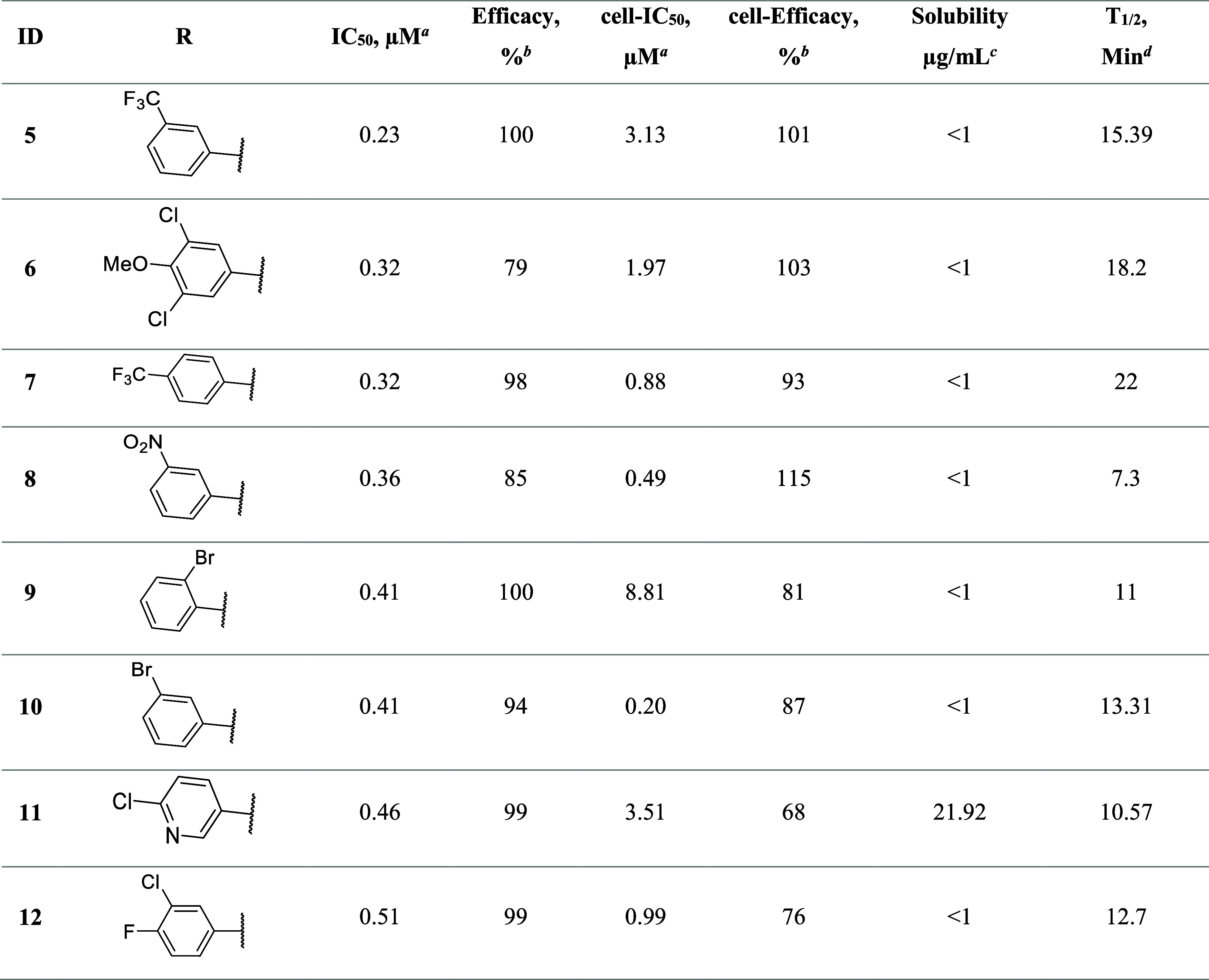
Most Potent Analogs in Biochemical Assay with Variation Around Piperazinyl Substituent, Their Activity in Cell-Based Assay, and Pharmacokinetic Properties

a IC_50_: half-maximal inhibitory concentration values obtained in 11-point dose response, measured in triplicate.

b Efficacy: maximum inhibitory effect observed in corresponding assay.

c Solubility-pION μSOL assay for kinetic aqueous solubility determination, pH7.4.

d *T*_1/2_: metabolic half-life measured in rat liver microsome fractions reported in minutes, a minimum detectable half-life of 1 min.

Next, we aimed to determine if the activity observed against isolated ALDH3A1 translated into a cellular environment using the 1,536-well ALDEFLUOR assay.^30^ Briefly, the ALDFLUOR substrate BAAA gets converted by ALDHs inside the cell to BAA, a cell-impermeable fluorescent product that labels cells with ALDH activity. BAAA is catalyzed by multiple ALDHs, with ALDH3A1 being among the weakest to process the substrate.^31–33^ With that caveat, we were still able to identify the OE19 cell line with preferential ALDH3A1 expression to assess inhibitors’ cellular activity^11^ (see Experimental Section). We tested all 72 synthesized analogs, with 53 compounds (73%) demonstrating cellular inhibition, exhibiting IC_50_ below 10 μM and efficacy values greater than 50% (Figure 4 (B); Supporting Information Table S4). Notably, compound **5**, which demonstrated potent biochemical inhibition (IC_50_ = 229 nM) and strong cellular activity (IC_50_ = 3.13 μM), was selected as the starting point for further optimization (Figure 4 (C)).

These findings indicate that a substantial proportion of the analogs achieved sufficient intracellular exposure, underscoring the potential of this scaffold series and highlighting strong starting point for further lead optimization.^34^

### CSAR Phase II: Reaction-Based Enumeration at R_2_

Following the success of the initial SAR campaign at the R_1_ position, we next extended our exploration to the R_2_ position of compound **5** to further improve the overall activity of the scaffold. Using the *in-silico* reaction enumeration strategy (Scheme 2), we generated approximately 250,000 analogs via virtual amide coupling reaction with commercially available amines.

**Scheme 2:**
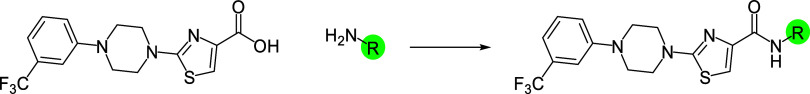
Amide Formation between Thiazole Carboxylic Acid Intermediate and Primary Amines^a^

a Reaction conditions used for synthesis: *N*-methylimidazole, TCFH, MeCN, r.t., 30 min.

To support compound prioritization, we retrained our QSAR model using updated biochemical data from the R_1_ substitution campaign. While model performance remained consistent overall with *R* ^2^ = 0.74 for 5-fold external cross validation procedure, the refined model offered improved resolution for ranking within the newly enumerated chemical space. From the total set of 251,225 analogs, 40,664 compounds were initially prioritized based on QSAR scores. We next performed pharmacophore-based molecular docking on this intersected set to assess compatibility with the ALDH3A1 binding site and filter compounds by docking score and pose alignment. From this evaluation, the top 700 analogs were shortlisted based on a combination of QSAR model rank, docking score, and binding pose quality. In close collaboration with medicinal chemists, we selected 85 prospective molecules for synthesis based on the combination of predictive score, cost and estimated delivery time of the building blocks, of which 78 analogs were successfully synthesized using standard amide coupling protocols (Scheme 2). Representative active analogs from this R_2_ optimization campaign, including the lead compounds NCATS-SM0707 and NCATS-SM0708, are summarized in Table 3.

**Table 3:**
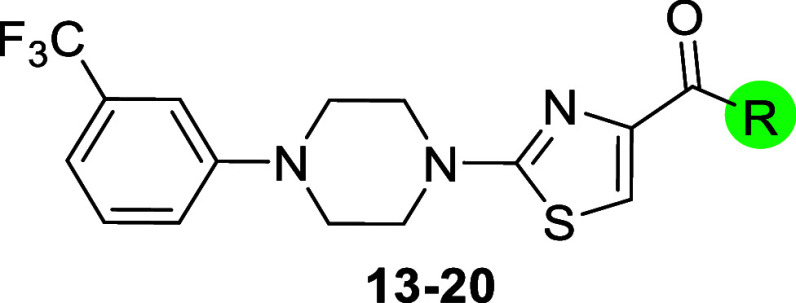

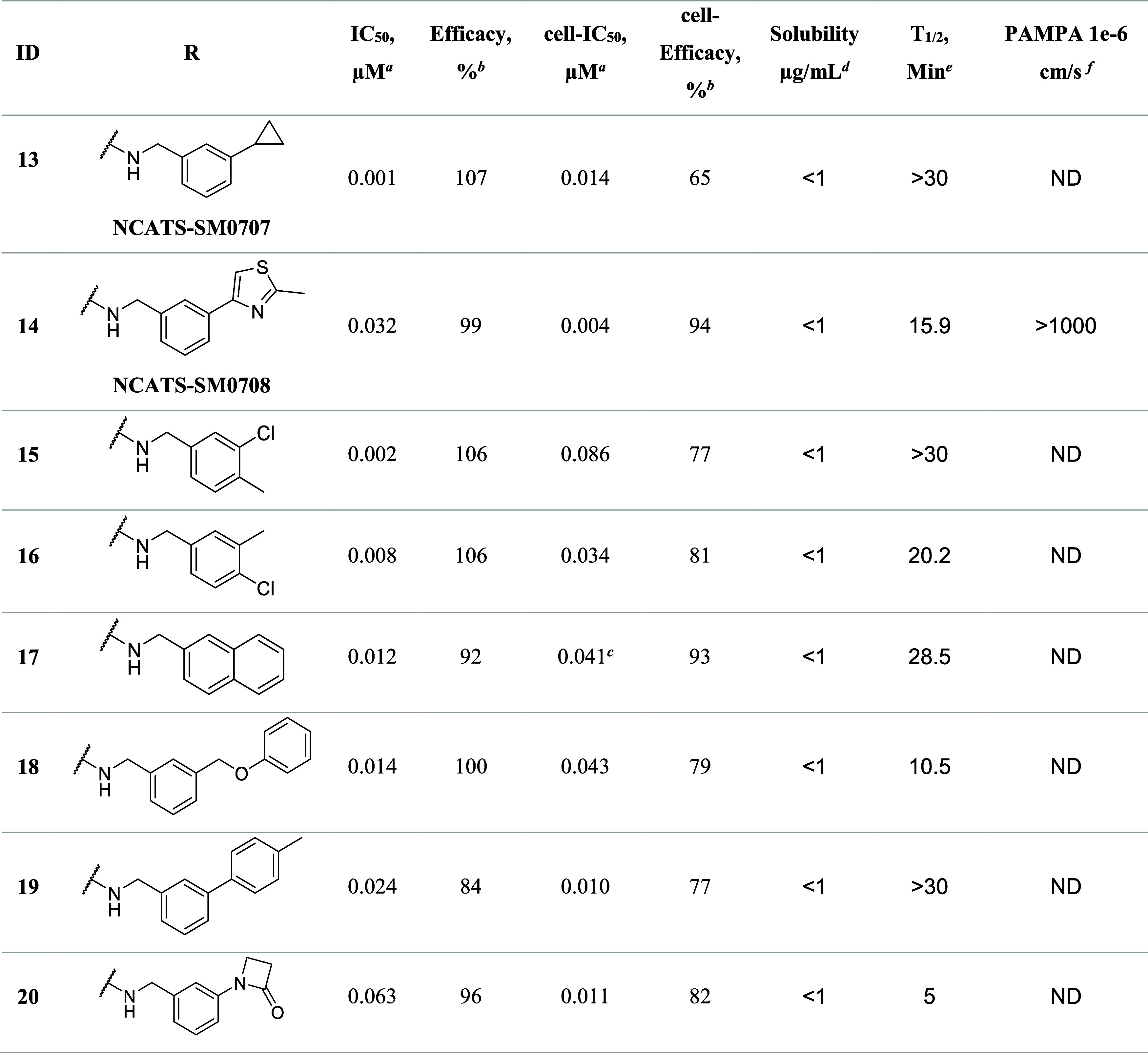
Representative Amine Substituents of the Notably Active Analogs at the Amide Site and Their Pharmacokinetic Properties

a IC_50_: half-maximal inhibitory concentration values obtained in 22-point dose response, measured in triplicate in biochemical assay and singleton in cell-based assay.

b Efficacy: maximum inhibitory effect observed in corresponding assay.

c Value represents data measured in triplicate.

d Solubility-pION μSOL assay for kinetic aqueous solubility determination, pH7.4.

e *T*_1/2_: metabolic half-life measured in rat liver microsome fractions reported in minutes, a minimum detectable half-life of 1 min.

f PAMPA (parallel artificial membrane permeation assay) is reported as a metric of the passive permeability of the compounds.

All 78 analogs were evaluated in parallel in both biochemical and cell-based ALDH3A1 assays. Biochemical testing revealed 72 compounds (92%) exhibited IC_50_ values below 20 μM, with 8 compounds showing sub-10 nM potency (Supporting Information Table S5). Using the best R_1_ analog **5** based on biochemical potency (IC_50_ of 229 nM) as a benchmark, 43 R_2_ analogs were more potent, underscoring the scaffold’s tractability for SAR optimization (Figure 5(A)). In the cell-based assay, 57 analogs showed cellular IC_50_ values below 10 μM, with several achieving submicromolar potency (Supporting Information Table S5). The activity distributions indicate that the R_2_-enumeration drastically improved cellular potency compared with R_1_ enumeration, with biochemical and cellular IC_50_ showing good overall correlation (Figure 5(B)). Figure 5(C) shows the spread of biochemical versus cellular activity for compounds generated across both CSAR optimization rounds. As illustrated, Round 1 analogs (blue) cluster tightly around moderate biochemical activity with modest cellular translation, whereas Round 2 analogs (red) reveal an expanded potency distribution, including multiple low-nanomolar inhibitors. This trajectory reflects the added benefit of R_2_-focused SAR exploration combined with predictive model refinement. When considering activity across both assay formats, compounds **13 (NCATS-SM0707)** and **14 (NCATS-SM0708)** emerged as the most promising candidate inhibitors (Table 3; Figures 5(D) and (E), respectively). These compounds demonstrated the highest potency in both the enzymatic assay (IC_50_ values of 1.12 nM and 31.63 nM) and the cellular ALDEFLUOR assay (IC_50_ values of 13.62 nM and 3.84 nM, respectively).

**Figure 5:**
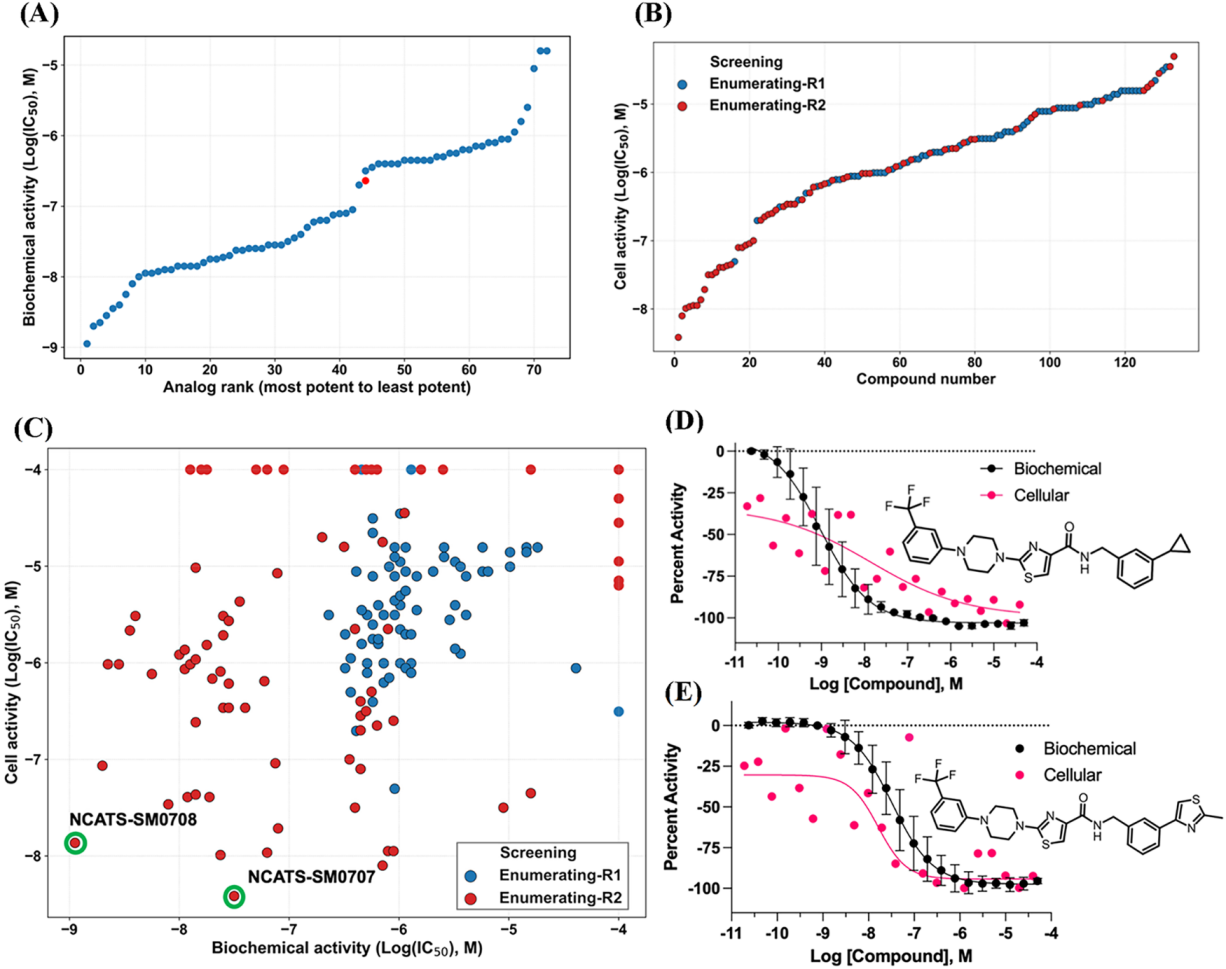
Reaction-based enumeration leads to potency gains through SAR refinement at R_2_. (A) Biochemical potency (log_10_[IC_50_], μM) of 78 analogs synthesized during Round 2 enumeration, ranked from most to least potent. The most active R_1_-derived analog (red) is highlighted for comparison. Multiple R_2_ analogs exceeded prior activity benchmarks, including nanomolar hits. (B) Cellular potency (log_10_[IC_50_], μM) of all synthesized compounds across both R_1_ (blue) and R_2_ (red) optimization rounds, showing relative distributions across the CSAR pipeline. (C) Correlation of biochemical and cellular activity across all analogs. R_1_ enumeration (blue) yields a tighter potency band, while R_2_ analogs (red) span a broader range with deeper biochemical and cellular translation, including multiple low-nanomolar inhibitors. The two most potent hits are highlighted (green circles). (D, E) Dose–response curves for the two most potent inhibitors: **NCATS-SM0707** (D) and **NCATS-SM0708** (E), with biochemical (black) and cellular (pink) activity plotted. Structures shown in inset. **NCATS-SM0707** achieved 1.12 nM biochemical and 13.62 nM cellular potency, while **NCATS-SM0708** (R_2_) exhibited 31.63 nM biochemical and 3.84 nM cellular activity.

### Selectivity and Binding Characterization of Candidate Inhibitors

We characterized the isoform selectivity of our lead compounds in biochemical assays against a panel of closely related ALDH isozymes, including ALDH1A1, ALDH1A2, ALDH1A3, and ALDH2, along with the closely related ALDH3A2 (∼70% sequence identity to ALDH3A1; see Experimental Section). In these assays, both **NCATS-SM0707** and **NCATS-SM0708** demonstrated excellent selectivity (defined as exhibiting >30-fold selectivity within a target family).^35^ **NCATS-SM0707** was weak to inactive against ALDH1A1, ALDH1A2, ALDH2, and ALDH3A2, exhibiting moderate activity against ALDH1A3 (IC_50_ = 3.16 μM, Efficacy = −60%), but still a strong >2,816-fold difference vs the ALDH3A1 activity (IC_50_ = 1.12 nM). Against the panel of ALDH-expressing cell lines, **NCATS-SM0707** was weak to inactive in OV90 (1A1), AN3CA (1A2) and PEO1 (1A3) cell lines (Figure 6(A)). **NCATS-SM0708** demonstrated similar bioactivity, with weak to inactive activity against ALDH1A1, ALDH1A2, ALDH2, and ALDH3A2, exhibiting moderate activity against ALDH1A3 (IC_50_ = 1.12 μM, Efficacy = −62%), a > 25-fold difference than the ALDH3A1 potency value of 31.6 nM. Against the panel of ALDH-expressing cell lines, **NCATS-SM0707** was inactive in AN3CA (1A2) cell line, exhibiting moderate activity vs OV90 (1A1; 0.562 μM, Efficacy = −56%) and PEO1 (1A3; 0.891, Efficacy = −57%), with IC_50_ ratios of 146 and 232, respectively, compared to the OE19 (3A1) potency value of 3.84 nM (Figure 6(B)). IC_50_ values were not reported for activity profiles for efficacies less than 40% or low-quality curve classes.

**Figure 6:**
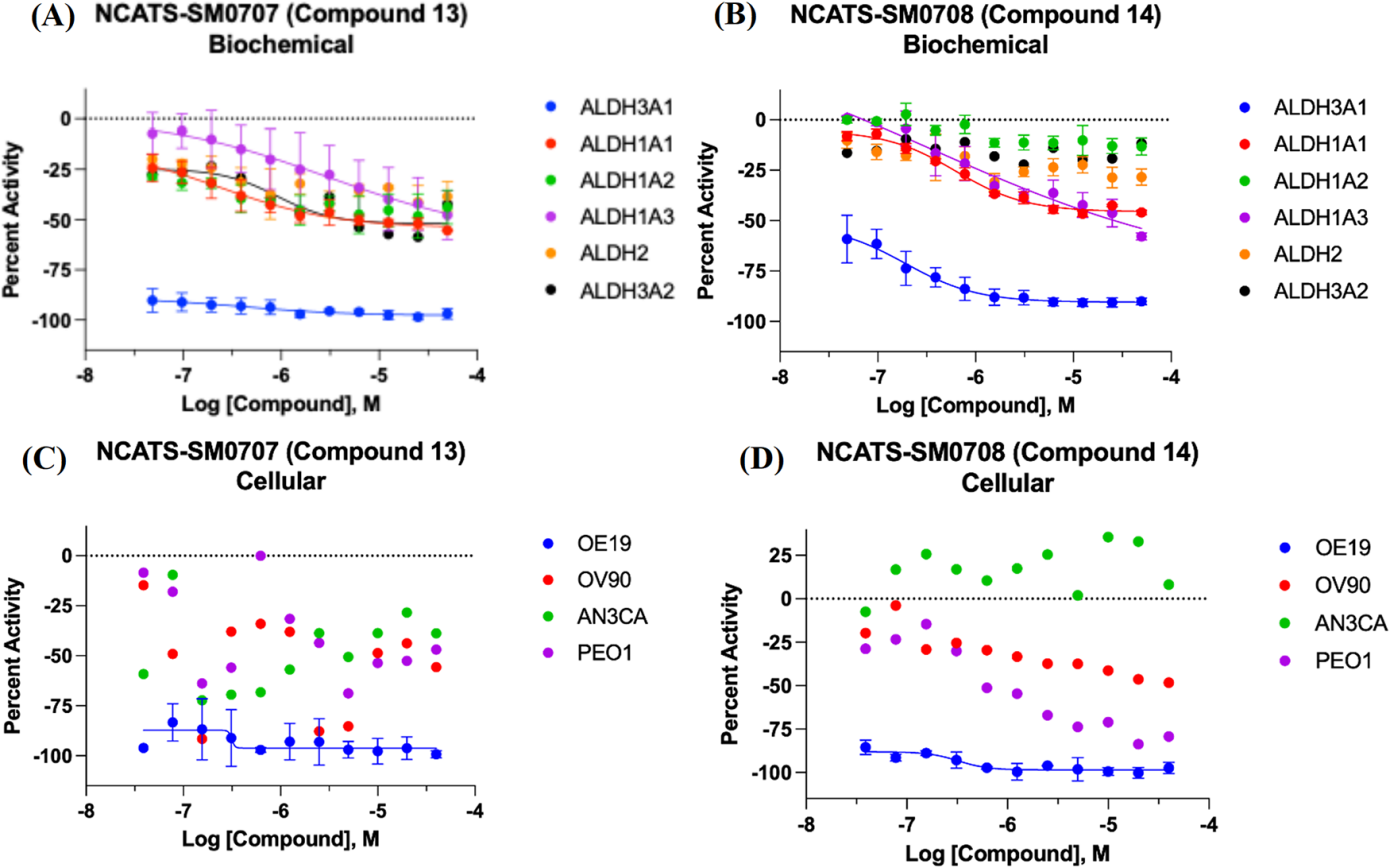
Selectivity profiling of ALDH3A1 inhibitors across ALDH isoforms and ALDH-expressing cell lines. (A, B) Biochemical IC_50_ values of **NCATS-SM0707** and **NCATS-SM0708** across a panel of ALDH isoforms demonstrate strong selectivity for ALDH3A1 over related family members, including ALDH1A1, ALDH1A2, ALDH1A3, ALDH2, and ALDH3A2. (C, D) Cellular profiling across multiple ALDH-expressing cell lines confirms this selectivity at the cellular level, with minimal off-target inhibition observed for other isoforms. Together, these data highlight the isoform specificity of both lead compounds, supporting their utility in probing ALDH3A1 biology with minimal cross-reactivity.

To gain preliminary insight into the inhibition mechanism of the lead compounds, we examined the effect of increasing benzaldehyde substrate concentration in the biochemical assay. In the standard assay format, benzaldehyde was tested at 200 μM, approximately 1× Km, whereas NAD­(P)+ was tested at 1000 μM, approximately 4× Km, to bias away from the cofactor pocket. To assess potential substrate competition, both **NCATS-SM0707** and **NCATS-SM0708** were retested at 4,000 μM benzaldehyde, corresponding to approximately 20× Km. Under these conditions, both compounds showed pronounced rightward shifts in their concentration–response curves (Supporting Information Figure S1 and Table S6), consistent with substrate-competitive inhibition. Because the assay was not configured to evaluate cofactor competition directly, additional kinetic studies will be required to distinguish fully between substrate-competitive and possible mixed modes of inhibition.

Finally, to confirm the binding of the compound to ALDH3A1, we synthesized a diazirine -containing analog **21** (Supporting Information Figure S2­(A)). Gratifyingly, analog **21** exhibited a potency profile comparable to that of the parent compound, demonstrating robust activity in both the ALDH3A1 biochemical assay (IC_50_ = 0.738 μM) and the cellular assay (IC_50_ = 15 nM) (Supporting Information Figure S2­(B)). For photo-cross-linking studies, analog **21** was preincubated with recombinant ALDH3A1 and subsequently irradiated with UV light to induce covalent cross-linking. The labeled ALDH3A1 protein was then digested and analyzed using an HPLC–MS/MS–based proteomics workflow. Diazirine-mediated photo-cross-linking resulted in a mass shift of 554.19 Da, for a specific modification of the amino acids at the peptide sequence WNAYYEEVVYVLE (Supporting Information Figure S2­(C)), which was further confirmed by competitive assay and control samples. This peptide sequence is a cluster of modified residues on an α-helical segment lining the ALDH3A1 active site; when projected onto the co-crystal structure to generate a computational model, these residues lie near the bound reference ligand, supporting that compound **13** engages the canonical substrate-binding pocket (Figure 7, Supporting Information Figure S2­(D)). Together, these data provide orthogonal support for direct ALDH3A1 engagement by this chemical series.

**Figure 7:**
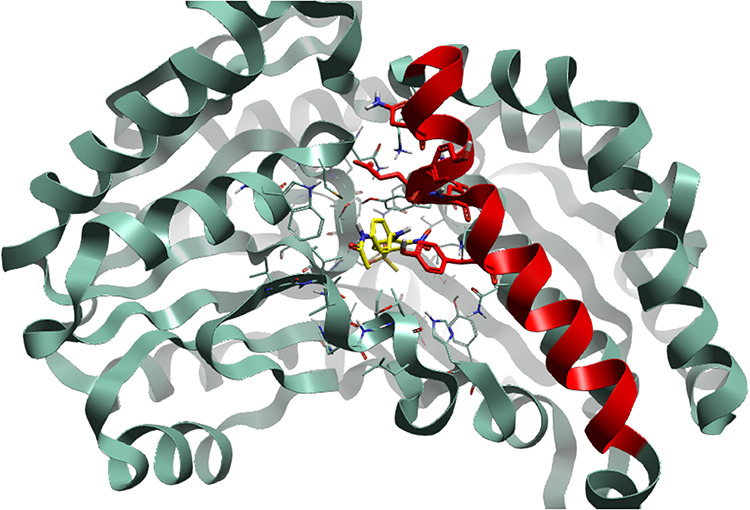
Structural mapping of the ALDH3A1 binding site using mass spectrometry. ALDH3A1 is shown as a teal ribbon with the cocrystallized reference ligand displayed as yellow sticks. Residues identified by mass spectrometry as modified in the presence of compound **13** (**NCATS-SM0707**) are highlighted in red on an α-helical segment lining the active site. Their proximity to the co-crystal ligand supports engagement of the canonical substrate-binding pocket by **NCATS-SM0707**.

## Discussion and Conclusion

The discovery of isoform-selective small-molecule inhibitors for therapeutically relevant yet chemically intractable targets remains a significant bottleneck in drug discovery.^36^ Traditional medicinal chemistry approaches, while effective, often operate through slow, iterative optimization cycles and are limited in their ability to explore chemical space broadly and efficiently.^11,37^ Here, we introduce and apply a modular, comprehensive SAR (CSAR) platform that integrates pharmacophore-based docking, scalable reaction enumeration, and predictive modeling to overcome these limitations, enabling rapid and systematic exploration of large synthetically tractable chemical space. We applied this workflow to ALDH3A1, a cancer-relevant enzyme for which potent and selective small-molecule inhibitors have been lacking.^38^ The results demonstrate the utility and broader applicability of this platform for accelerating hit-to-lead optimization, particularly for targets where conventional approaches have yielded limited progress.

The advantage of this CSAR approach lies in its ability to merge the logic of medicinal chemistry with the scale and speed of modern computational tools. Starting from a validated hit, we used reaction-based enumeration to explore substituent space in a way that mirrors traditional R-group optimization strategies (e.g., R_1_ to R_2_), but at a significantly larger scale, spreading across a larger chemical space. By combining commercially available building blocks with predictive modeling, we were able to generate and triage ∼250,000 virtual analogs across two focused enumeration campaigns. This level of exploration would be difficult to achieve through manual design or catalog-based selection alone. Importantly, the high synthesis success rates in both rounds (90% and 100%) reflect the practical feasibility built into the enumeration process, an often underappreciated aspect in in silico design workflows. It is also important to note that the framework itself is not limited to the two transformations demonstrated here. In principle, it can be extended through additional reaction schemes and iterative design cycles to incorporate broader medicinal chemistry modifications, including linker variation, scaffold diversification, and related chemotype-level changes when required by the optimization campaign.

Our case study with ALDH3A1 demonstrates how this platform can accelerate SAR development around a relatively unexplored target. Although ALDH3A1 has been implicated in chemoresistance and immune evasion, progress toward selective chemical probes has been limited by the lack of potent and isoform-specific inhibitors. Our initial pharmacophore-based virtual screen identified a thiazole-containing sulfonamide scaffold with suitable tractability and binding characteristics. Using this as the starting point, we conducted two successive rounds of reaction-based enumeration—first at R_1_, then R_2_—to systematically explore SAR. The R_1_ campaign delivered a high hit rate (97% with IC_50_ < 30 μM) and clear improvements in potency, with multiple analogs reaching sub-230 nM in biochemical assays and <5 μM in cells. The speed at which these optimizations were achieved, within a single design round, highlights the practical value of incorporating predictive models early in the compound selection process.

The second round of optimization, focused on the R_2_ position of the hit scaffold, led to further gains in both biochemical and cellular potency. For this phase, we utilized the updated QSAR model based on the deep learning consensus architecture (DLCA), allowing us to further prioritize compounds using complementary predictive strategies. The combination of models built on distinct molecular representations helped identify high-confidence candidates from a large enumerated library. This approach led to the synthesis of most active analogs, including compound **13 (NCATS-SM0707)** and **14 (NCATS-SM0708)**, which achieved 1 nM and 32 nM biochemical potency as well as 14 nM and 4 nM cellular activity, respectively. We further validated the binding mode based on a diazirine photoaffinity analog **21** using proteomics approach. The results provide direct evidence that the compounds engage ALDH3A1 within the canonical substrate-binding pocket, supporting the proposed mechanism of action derived from biochemical and structural analyses. With excellent cellular activity, initial in vitro ADME profiling, and isoform selectivity, the compounds demonstrate a significant advance over previously reported ALDH3A1 inhibitors (e.g., CB7), emerging as strong ALDH3A1 leads for further optimization and as effective tools for validating ALDH3A1 as a therapeutic target in cancer models.

The particularly strong cellular activity of **NCATS-SM0708** (4 nM) further highlights the ability of this workflow to deliver compounds that translate potent ALDH3A1 inhibition into a whole-cell setting. This result supports the value of the series for further biological investigation and continued lead optimization. At the same time, despite the strong biochemical potency, cellular activity, and isoform selectivity achieved in this series, the current leads still retain some important limitations for further development. In particular, many of the most potent analogs, including **NCATS-SM0707** and **NCATS-SM0708**, showed limited kinetic aqueous solubility, and **NCATS-SM0708** also displayed only moderate microsomal stability. While these liabilities are partly offset by the strong potency achieved in the series, the compounds are best viewed as potent and selective lead compounds rather than development-ready candidates. Future iterations of the workflow should incorporate multiparameter optimization criteria, particularly solubility and metabolic stability, in addition to potency and selectivity.

From a medicinal chemistry perspective, hit-to-lead efficiency metrics provided additional insight into the optimization trajectory of the starting hit and the two lead compounds (Supporting Information Table S7). Notably, **NCATS-SM0707** improved both ligand efficiency and lipophilic ligand efficiency relative to the initial hit, indicating that the marked potency gain was not achieved solely through increased molecular size or lipophilicity. In contrast, **NCATS-SM0708** retained strong potency in both biochemical and cellular assays but showed lower efficiency metrics, highlighting a different balance between potency and physicochemical cost in this branch of the series.

To better understand the structural basis of potency improvement during optimization, we compared docked poses of the original hit and **NCATS-SM0707** within the ALDH3A1 binding pocket (Supporting Information Figure S3). The analysis showed that the optimized analog remained compatible with the same general binding region as the original scaffold and preserved the core placement of the thiazole-amide framework within the substrate-binding pocket. Notably, **NCATS-SM0707** yielded two similarly plausible docking orientations, including one aligned with the original hit pose and another flipped orientation, indicating that the precise binding geometry cannot be assigned unambiguously from docking alone. Although these models did not identify a single clearly gained interaction at the R_2_ position sufficient to account for the full potency increase, they support improved overall complementarity of the optimized analog within the pocket while retaining productive engagement of the canonical binding region. A more detailed mechanistic dissection of the energetic basis of this gain would require higher-resolution structural or biophysical studies beyond the scope of the present work.

The reliability of our QSAR-based predictions was strengthened by applying an applicability domain filter based on Tanimoto similarity calculated using Morgan fingerprints.^29,39^ By comparing each enumerated compound to the original training set and enforcing a similarity threshold of 0.6, we constrained the prediction space to regions of chemistry the model was trained on. This step limits the predictions to the model’s chemical space, thereby improving reliability and minimizing uncertainty associated with extrapolation. In practice, the applicability domain filter increased the robustness of the triage process and helped focus attention on analogs most likely to translate into measurable biological activity. As a result, it served as an important refinement layer that balanced prediction-driven exploration with chemical relevance and experimental feasibility.

The isoform selectivity profile of **NCATS-SM0707** and **NCATS-SM0708** significantly offers its value as a lead for probing ALDH3A1 biology. Within the ALDH enzyme family, achieving selectivity is a known challenge due to substantial sequence and active-site homology.^36^ The isoform selectivity of our lead compounds **NCATS-SM0707** and **NCATS-SM0708** is readily explained by our ALDH3A1-centric triage approach. Our pharmacophore hypothesis and docking filters were built on the ALDH3A1 binding site, rewarding complementarity to its pocket volume, and hydrogen-bond topology that are not conserved across the ALDH family. This design biases advancement toward chemotypes that fit 3A1 and away from motifs preferred by the counter-isoforms. In both biochemical and cell-based assays, both compounds **NCATS-SM0707** and **NCATS-SM0708** demonstrated excellent selectivity for ALDH3A1 over other closely related isoforms, including ALDH1A1, ALDH1A2, ALDH2, and ALDH3A2. This high degree of selectivity reduces the likelihood of off-target effects and enables confident assignment of biological outcomes to ALDH3A1 inhibition specifically.

The developed platform can be extended beyond the specific two-round enumeration demonstrated here, as it is designed to be highly adaptable by incorporating diverse chemical reactions at various molecular positions to explore a virtually unlimited chemical space. While our CSAR platform offers significant advantages, it is important to acknowledge its inherent limitations. The quality of the enumerated library is dependent on the availability and diversity of commercial building blocks and the feasibility of the reaction templates. Similarly, the predictive power of the QSAR models is intrinsically linked to the quantity and quality of the experimental training data.^40^ Future improvements could involve integrating more advanced generative models for scaffold hopping alongside SAR expansion, as well as incorporating ADMET (absorption, distribution, metabolism, excretion, and toxicity) predictions earlier in the design pipeline to further refine compound prioritization.

In conclusion, our work demonstrates a structured, scalable, and generalizable AI-guided complementary and comprehensive hit-to-lead platform for accelerating the drug discovery process. By integrating pharmacophore-based docking, reaction-based enumeration, and machine learning, our CSAR approach bridges the gap between traditional medicinal chemistry intuition and the vastness of computationally accessible chemical space. The successful identification of a highly potent, cell-active, and isoform-selective ALDH3A1 inhibitors represents a significant advance for the biological study of ALDH3A1 and underscores the transformative potential of AI-driven strategies in modern drug discovery. This workflow offers a practical and adaptable strategy for advancing challenging therapeutic targets, enabling the discovery of high-quality chemical probes and guiding the development of future therapeutics

## Experimental Section

### Pharmacophore-Based Docking and Virtual Screening

Pharmacophore-based molecular docking was performed using the Molecular Operating Environment (MOE) software.^41–43^ The crystal structure of human ALDH3A1 (PDB ID: 4H80) was used to define the ligand-binding site and to derive a structure-based pharmacophore model. This model included essential interaction features observed in the cocrystallized ligand, such as hydrogen bond donors and acceptors, hydrophobic regions, and aromatic rings, critical for molecular interactions. The ligand-binding pocket in the cocrystal structure of ALDH3A1 (PDB ID: 4H80)^38^ was used as the active site for all docking studies. Protein preparation was performed using MOE. The steps include removal of water molecules and irrelevant heteroatoms, addition of hydrogens, assignment of appropriate protonation states, and energy minimization to optimize the protein conformation for docking.^44,45^ The compound library for docking comprised approximately 10,000 structurally diverse molecules. It was prepared using MOE’s ligand preparation module, which includes 3D conformation generation, protonation state assignment, and energy minimization to ensure docking-ready structures.

### Docking Protocol

The docking protocol involved aligning the compounds from the prepared library to the pharmacophore model to identify potential binders.^46–48^ MOE’s docking algorithm (Affinity dG scoring function) was used to simulate the interaction between each compound and the target binding site. Postdocking, each compound was scored based on its docking pose and the degree of match with the pharmacophore model. The scoring function in MOE provided a quantitative measure of the binding affinity. Compounds were then ranked based on their scores, with higher-scoring compounds considered more likely to be active.

### Reaction-Based Compound Enumeration with KNIME

Reaction-based enumeration was performed using the KNIME Analytics Platform,^49,50^ which provided a flexible environment for building and executing cheminformatics workflows.^51^ Our approach focused on two key synthetic reactions: Amide coupling with various amines and nucleophilic aromatic substitution with *N*-substituted piperazines to explore chemical space around the core scaffold. We generated the reaction SMARTS for the two reactions and, using the RDKit two-component reaction node within KNIME,^52^ systematically generated a virtual library of compounds by combining the selected scaffold with commercial building blocks from Enamine. The reaction enumeration was conducted in two phases. The first focused on substituent variation at the R_1_ position, generating 9336 analogs. The second phase targeted the R_2_ position and yielded about 250,000 additional compounds. This strategy allowed systematic exploration of substitution patterns across both sides of the scaffold, supporting SAR development and identification of tractable analogs for synthesis.

To prioritize compounds for further study, a QSAR model trained on internal biochemical screening data was applied to the enumerated libraries. This enabled selection of candidates with favorable predicted activity prior to docking and synthesis.

### Quantitative Structure–Activity Relationship (QSAR) Modeling for Compound Prioritization

We developed and applied a deep learning consensus architecture (DLCA) model^28^ for the rank order of R_1_ and R_2_ enumerated sets. DLCA was previously showed similar or better predictive results compared to the modern state-of-the-art machine learning and deep learning methods on both classification and regression tasks.^28,29^ This framework integrates predictions from multiple deep neural networks trained on diverse molecular representations, including Morgan, Avalon, and AtomPair fingerprints, RDKit physicochemical descriptors, and a convolutional neural network built on SMILES strings.^29^ This architecture can be easily extended by including other types of molecular descriptors and/or molecular representations (e.g., molecular graphs). Final predictions were derived by averaging outputs from these networks, providing a consensus score that balances the strengths of each representation while reducing error propagation. We also developed Random Forest^53^ models using RDKit physicochemical descriptors, Morgan, Avalon, and AtomPair fingerprints, along with their combined feature set, for comparison with the DLCA model.

To ensure reliable prediction from our QSAR model’s, we applied the applicability domain criterion using Tanimoto similarity calculated using Morgan fingerprints.^28,54,55^ Compounds with a similarity score of at least 0.6 to those in the training set were selected, keeping the analysis within chemical space closely related to known actives while preserving sufficient coverage of the reaction-defined analog space for prospective prioritization. Batch balancing was performed for regression task to eliminate a prediction bias toward less active compounds.

This allowed us to confidently narrow down large virtual libraries to a focused set of candidates for synthesis and experimental validation.

### Enzymes

Human ALDH1A1, ALDH1A2 and ALDH3A1 recombinant enzymes were purchased from R&D Systems (Minneapolis, MN). Human ALDH1A3 recombinant enzyme was purchased from Sino Biological (Wayne, PA). Human ALDH2 recombinant enzyme was purchased from Abcam (Cambridge, MA). For ALDH3A2, a gateway entry clone for ALDH3A2(1–460) was synthesized by ATUM, Inc. and optimized for expression in *E. coli*. The Entry clone contained upstream tobacco etch virus (TEV) protease sites (ENLYFQ/G) and was subcloned into an expression clone using Gateway LR recombination into pDest-566 (Addgene #11517) to create *E. coli* T7-based expression vectors with a His6-MBP (maltose binding protein) N-terminal fusion tag. Final expression clone was validated by agarose gel electrophoresis and restriction digest.

Bacterial expression construct was transformed into *E. coli* BL21*­[pRare] cells and plated on LB agar with 100 μg/mL ampicillin and 15 μg/mL chloramphenicol at 37 °C. A seed culture was started from a single colony in 50 mL of MDAG-135 medium and grown overnight at 37 °C. Forty milliliters of seed culture was used to inoculate 2 L of Dynamite medium^56^ in a BioFlo 110 bioreactor (3-L vessel, New Brunswick). Cells were grown at 37 °C to an OD600 of 6–8, before adding 0.5 mM IPTG to induce protein expression at 16 °C for 19 h. Cells were collected by centrifugation at 4,000*g* for 20 min and frozen at −80 °C.

Cell pellet was thawed and resuspended in lysis buffer (20 mM HEPES, pH 7.4, 300 mM NaCl, 5 mM TCEP, 1:200 v:v Sigma Protease Inhibitor Cocktail #P8849) using a volume of 1 mL/100 OD600 units. Cells were lysed in a microfluidizer with 2 passes at 10,000 psi. Lysates were clarified by ultracentrifugation at 100,000*g* for 30 min at 4 °C, followed by filtration of the soluble fraction with a 0.45 μm PES filter. Chromatography was conducted at room temperature (∼22 ◦C) using NGC medium-pressure chromatography systems from BioRad Laboratories Inc. (Hercules, CA).

The lysate was adjusted to 35 mM imidazole and loaded onto equilibrated IMAC (immobilized metal affinity chromatography) columns (Ni Sepharose High Performance nickel-charged resin, Cytiva, Marlborough, MA) at a ratio of 20 mL of resin/L of culture. Column equilibration buffer was 20 mM HEPES, pH 7.4, 300 mM NaCl, 5 mM TCEP plus 35 mM imidazole; the imidazole is added to reduce nonspecific binding to the purification resin. The column was washed to baseline with the equilibration buffer. A 5 column-volume (CV) gradient was implemented to 500 mM imidazole, followed by a 2 CV wash of 500 mM imidazole. SDS-PAGE and Coomassie-staining were used for elution fraction analysis. Appropriate fractions were pooled, His6-TEV protease (purified in-house) at 4 mg/mL was added at a 1:20 (v/v), and the digestion pool dialyzed using 10 K MWCO membrane (SnakeSkin Dialysis Tubing, Thermo Fisher Scientific) against the equilibration buffer without imidazole overnight at 4 °C. Another IMAC was performed using the same Ni Sepharose High Performance nickel-charged resin (Cytiva, 20 mL of resin/L of culture), no imidazole was present in the equilibration or wash buffers. A 3 CV gradient was performed to 100 mM imidazole, followed by a 2 CV wash of 500 mM imidazole. Appropriate fractions were pooled after SDS-PAGE and Coomassie-staining analysis and concentrated using 10K MWCO Pellicon XL cassette (EMD Millipore) to an appropriate volume for size exclusion chromatography (SEC).

The concentrated pool was loaded onto an equilibrated HiLoad 16/600 Superdex 75 resin (Cytiva) column in final buffer of 20 mM HEPES, pH 7.4, 150 mM NaCl, 5 mM TCEP. Appropriate fractions were pooled using SDS-PAGE and Coomassie-staining analysis and concentrated if needed by a 10K MWCO Amicon centrifugation units (EMD Millipore) and filtered with a Millex-GP 0.22 μM syringe filter (EMD Millipore). The protein concentration was determined by measuring 280 nm (Nanodrop 2000C Spectrophotometer, Thermo Fisher Scientific), and snap frozen using liquid nitrogen in 50 μL aliquots in 1.5 mL Eppendorf tubes.

### Biochemical Assay for ALDH Inhibition

The assay has been previously published ALDH1A1, ALDH1A2, ALDH1A3, ALDH2, and ALDH3A1.^11^ All enzyme concentrations are nominal. When possible, substrate concentrations were kept above *K*_m_ to bias the assay for noncompetitive inhibitors. Briefly, 3 μL of enzyme (final concentration of 5 nM and 25 nM for ALDH3A1 and ALDH3A2, respectively) or assay buffer (100 mM HEPES pH 7.5 with 0.01% Tween 20) were dispensed into a 1,536-well black solid-bottom plate. Twenty nL of compounds (11- or 22-point dilutions, final concentration range 23.7 pM to 50 μM) or control (DEAB or ZM-39923 final concentration range 23.7 pM to 50 μM) were transferred via acoustic droplet ejection. Samples were incubated (room temperature, protected from light) for 15 min followed by an addition of 1 μL substrate mixture of NAD­(P)+ and benzaldehyde or propionaldehyde (final concentrations of 1 mM and 200 μM; 1 mM and 1,000 μM for ALDH3A1 and ALDH3A2, respectively; *K*_m_’s of 260 μM^57,58^ and 280 μM^57,58^ 8.7 mM and 1,300 μM for ALDH3A2),^59^ in addition to coupling reagents Resazurin and Diaphorase (final concentrations of 100 μM and 0.7 U/mL) to right-shift the detection by monitoring the production of resorufin.^60,61^ Plates were centrifuged at 1,000 rpm (164*g*) for 15 s, then read (RT) in kinetic mode on a ViewLux High-throughput CCD imager equipped with standard Rhodamine optics (525 nm excitation, 598 nm emission) for 5 min (<20% conversion). The change in fluorescence or luminescence intensity over the respective reaction period was normalized against no-inhibitor or no-enzyme for neutral (DMSO) or positive controls, respectively.

### Cell lines and Culture Conditions

SKBR3, AN3CA, and OV90 cells were obtained from America Type Culture Collection, (ATCC, Manassas, VA; #HTB-30, #HTB-111, #CRL-11732, respectively), PEO1 and OE19 cells were obtained from Sigma-Aldrich (MilliporeSigma, Rockville, MD; #10032308–1VL, #and #96071721–1VL, respectively). PEO1 and OE19 were cultured in RPMI 1640 (Life Technologies), supplemented with 2 mM l-Glutamine (Life Technologies), 10% HyClone fetal bovine serum (FBS, GE Healthcare) and 100 U/mL penicillin and 100 μg/mL streptomycin (referred to as 1% Pen/Strep, Life Technologies). SKBR3 were cultured in McCoy’s 5A (GIBCO) supplemented with 10% FBS and 1% Pen/Step. AN3CA were cultured in EMEM (ATCC) supplemented with 10% FBS and 1% Pen/Strep. OV90 were cultured in MCDB 105 (Cell Applications) and Medium 199 (ThermoFisher) supplemented with 15% FBS, 1.85 g/L sodium bicarbonate (ThermoFisher), and 1% Pen/Strep. All cell lines were maintained at 37 °C, 5% CO2, 85% RH, and routinely tested for mycoplasma contamination.

### Cellular ALDEFLUOR Activity Assays

ALDEFLUOR assays were performed as previously described.^11^ Briefly, OE19 cells (5 μL) were filtered (40 μm cell strainer) then dispensed into black, optical quality, clear bottom, TC treated 1,536-well plates (Aurora Microplates) at a density of 1,500 cells/well using a Multidrop Combi dispenser (ThermoFisher) and incubated overnight (37 °C, 5% CO2, 85% RH). Media was subsequently removed by centrifuging plates upside down using a plate adaptor to collect media. Five μL/well of a solution of 1,000 nM BAAA substrate (STEMCELL Technologies) and 0.5 nM (or 5 μg/mL) Hoechst 33342 (ThermoFisher) in ALDEFLUOR buffer (STEMCELL Technologies) was dispensed onto cells using a Multidrop Combi followed by immediate acoustic transfer of 20 nL compound or control solutions using an Echo 655 acoustic dispenser. The neutral and positive controls were DMSO (0.4% final) and DEAB (final concentration 40 μM). Compounds were assayed as 11- or 22-point dilutions spanning a final concentration range of 19 pM to 40 μM. Cells were incubated for 1 h at 37 °C to allow the conversion of BAAA into BAA. Supernatant was subsequently removed by centrifugation as described above, then ALDEFLUOR buffer (5 μL/well) was dispensed by Multidrop Combi before imaging. Images were captured on an Opera Phenix (PerkinElmer) widefield automated microscope using standard DAPI (390/18×, 432/48m) and FITC (475/28×, 525/48m) filter sets. Images were analyzed using Columbus image analysis system (PerkinElmer).

### qHTS Data Analysis and Statistics

Data from each assay were normalized plate-wise to corresponding intraplate controls as noted above. Concentration–response curves were fitted and classified as described previously.^62–66^ IC_50_’s were calculated from Prism software (version 10.4.2, GraphPad Software, Inc.) used sigmoidal dose–response (variable slope). All qHTS screening results are publicly available at https://pubchem.ncbi.nlm.nih.gov/source/NCGC.

### Photo-Cross-Linking and Mass Spectrometry

Recombinant human ALDH3A1 enzyme was concentrated in Amicon ultra centrifugal filter (Millipore, 10k MWCO) to a concentration of 1 mg/mL. The protein storage buffer was exchanged to 50 mM Tris, 150 mM NaCl, 5 mM DTT, 500 μM NAD­(P)+, pH 7.5 buffer using Zeba spin column (ThermoFisher, 7k MWCO). Sixteen μg ALDH3A1 protein was incubated with DMSO or compound 4042 (final concentrations of 25 or 50 μM) for 30 min at RT, then irradiated at ∼355 nm for 20 min on ice. All the samples were denatured and reduced with the addition of 0.1% RapidGest (Waters) and 15 mM Tris­(2-carboxyethyl)­phosphine (TCEP, Millipore Sigma) at 55 °C for 45 min, and alkylated with 15 mM iodoacetamide (IAA, Millipore Sigma) in the dark for 30 min. The alkylated proteins were then precipitated by adding 9-fold volumes of cold acetonitrile (ACN, Thermo Fisher) and incubated on ice for 15 min, followed by centrifugation at 14,000 g for 10 min at 4 °C. After aspirating the supernatant, the proteins were redissolved in trypsin/Lys-C (Progema V507A) buffer in 25 mM ammonium bicarbonate (NH_4_HCO_3_, Millipore Sigma, pH 8.0) at an enzyme/substrate ratio (m/m) of 1:50 and incubated at 37 °C overnight. The digestion was quenched by adding 1% of formic acid (FA, Thermo Scientific) and the supernatant was transferred to a clean and protein low-bind tube for further LC-MS/MS analysis.

### LC-MS/MS and Data Analysis

LC-MS/MS analysis of the protein digest was performed using an UltiMate 3000-nano LC system coupled to the Orbitrap Fusion Lumos Tribrid mass spectrometer equipped with the Nanospray Flex ion source (Thermo Fisher). Peptides were loaded onto the trap column (AcclaimPepMap 100 C18, 75 μm × 2 cm, particle size: 3 μm, 100 Å; Thermo Fisher) by autosampler using loading solvent (2% ACN in 97.9% MS-grade water, 0.1% FA) at a flow rate of 4 μL/min. Elution of peptides from the analytical column (Aurora Ultimate XT 25 cm × 75 μm, 1.7 μm, C18; IonOpticks, Australia) was performed using a 70 min method (54 min gradient) starting at 98% buffer A (0.1% FA in MS-grade water) at a flow rate of 400 nL/min. The mobile phase was maintained at 2% buffer B (80% ACN, 19.9% water, 0.1% formic acid) for 5 min, 2–6% B for 0.5 min, 6–31% B for 38 min, 31–43% B for 6.5 min, 43–95% B for 1 min, and maintained at 95% B for 8 min, followed by re-equilibration of the column with 2% B for 10 min. Column oven temperature was set as 50 °C.

The mass spectrometer was operated in positive-ionization mode with spray voltage set at 1800 V, and ion transfer tube temperature set at 300 °C. The MS scan was operated at data-dependent acquisition mode, with full MS scans over a mass range of *m*/*z* 375–1800 with detection in the Orbitrap (120 K resolution) and with auto gain control (AGC) set to 1.0 × 10^6^. The fragment ion spectra were acquired in Orbitrap (15 K resolution) with a normalized collision energy of 28% at HCD activation mode. In each cycle of data-dependent acquisition analysis, the most intense ions were selected for the MS/MS analysis, and the cycle time for MS and MS/MS analysis was set as 2 s. The AGC for MS/MS was set as 5.0 × 10^4^ and the maximum injection time was 22 ms. Precursor ions with charges of +2 to +7 were isolated for MS/MS sequencing. The MS/MS isolation window was 1.2 Da, and the dynamic exclusion time was set at 60 s with a mass tolerance of ± 10 ppm.

Proteome Discoverer software suite (version 3.1.1.93, Thermo Fisher) with Sequest algorithm was used for peptide identification. The MS raw data were searched against a Swiss-Prot human database (version Nov 2022, reviewed database) consisting of 20,328 entries using the following parameters: precursor ion mass tolerance of 10 ppm and a fragment ion mass tolerance of 0.02 Da. Peptides were searched using fully tryptic cleavage constraints and up to two internal cleavage sites were allowed for tryptic digestion. Variable modifications considered were carbamidomethylation of cysteine, oxidation of methionine residues, deamidation of asparagine, and compound 21 adduct (+554.1963) of any amino acid residue.

### Compound Synthesis and Characterization

Compounds **1**-**4** represent analogs from the internal library collection.

#### General Procedure **A**

To the solution of 2-chloro-*N*-(3-methylbenzyl)­thiazole-4-carboxamide (0.054 g, 0.20 mmol) in DMSO (0.2 M, 1 mL) was added *N*-substituted piperazine (1 equiv), and the reaction mixture was stirred at 110 °C for 6 h. Upon completion the mixture was filtered, and the filtrate was purified on a preparative HPLC to obtain pure products after lyophilization.

#### General Procedure **B**

To a mixture of 2-(4-(3-(trifluoromethyl)­phenyl)­piperazin-1-yl)­thiazole-4-carboxylic acid (0.04 g, 0.11 mmol) and primary amine (1 equiv) in MeCN (0.2 M, 0.560 mL) was added 1-methyl-1H-imidazole (4.5 equiv., 0.040 mL, 0.504 mmol) followed by TCFH (1.2 equiv., 0.038 g, 0.134 mmol), and the reaction mixture was stirred for 30 min at room temperature. The excess solvent was evaporated, the crude residue was redissolved in DMSO, and the filtrate was purified on a preparative HPLC to obtain pure products after lyophilization.

Compounds **5–12** were prepared according to the *General Procedure A*.

##### Compound **5**

^1^H NMR (400 MHz, DMSO-*d*_6_) δ 8.61 (t, *J* = 6.4 Hz, 1H), 7.51–7.41 (m, 2H), 7.30 (dd, *J* = 8.4, 2.5 Hz, 1H), 7.25 (s, 1H), 7.20 (dd, *J* = 7.5, 7.5 Hz, 1H), 7.15–7.01 (m, 4H), 4.39 (d, *J* = 6.4 Hz, 2H), 3.66–3.57 (m, 4H), 3.41–3.38 (m, 4H), 2.28 (s, 3H). LCMS (Method 1) t_r_ = 6.81 min, *m*/*z* (M + H)^+^ = 461.1.

##### Compound **6**

^1^H NMR (500 MHz, Chloroform-*d*) δ 7.51–7.43 (m, 2H), 7.25–7.22 (m, 1H, overlaps with chloroform peak), 7.18–7.13 (m, 2H), 7.10 (d, *J* = 7.5 Hz, 1H), 6.85 (s, 2H), 4.58 (d, *J* = 6.1 Hz, 2H), 3.84 (s, 3H), 3.65–3.55 (m, 4H), 3.26–3.17 (m, 4H), 2.35 (s, 3H). LCMS (Method 1) t_r_ = 6.94 min, *m*/*z* (M + H)^+^ = 491.1.

##### Compound **7**

^1^H NMR (400 MHz, DMSO-*d*_6_) δ 8.61 (t, *J* = 6.4 Hz, 1H), 7.52 (d, *J* = 8.6 Hz, 2H), 7.45 (s, 1H), 7.18 (dd, *J* = 7.5, 7.5 Hz, 1H), 7.12 (d, *J* = 8.5 Hz, 2H), 7.09–6.98 (m, 3H), 4.37 (d, *J* = 6.4 Hz, 2H), 3.64–3.56 (m, 4H), 3.47–3.39 (m, 4H), 2.26 (s, 3H). LC-MS (Method 1) t_r_ = 6.74 min (M + H)^+^ = 461.2.

##### Compound **8**

^1^H NMR (400 MHz, DMSO-*d*_6_) δ 8.62 (t, *J* = 6.4 Hz, 1H), 7.73 (t, *J* = 2.3 Hz, 1H), 7.63 (dt, *J* = 7.5, 1.7 Hz, 1H), 7.56–7.44 (m, 3H), 7.20 (t, *J* = 7.5 Hz, 1H), 7.13–7.01 (m, 3H), 4.39 (d, *J* = 6.3 Hz, 2H), 3.67–3.60 (m, 4H), 3.48–3.41 (m, 4H), 2.28 (s, 3H). LCMS (Method 1) t_r_ = 6.24 min, *m*/*z* (M + H)^+^ = 438.2.

##### Compound **9**

^1^H NMR (500 MHz, Chloroform-*d*) δ 7.59 (dd, *J* = 7.9, 1.5 Hz, 1H), 7.51 (q, *J* = 4.7 Hz, 1H), 7.46 (s, 1H), 7.29 (td, *J* = 7.7, 1.5 Hz, 1H), 7.24 (dd, *J* = 7.5, 7.5 Hz, 1H), 7.19–7.13 (m, 2H), 7.10 (d, *J* = 7.5 Hz, 1H), 7.05 (dd, *J* = 8.0, 1.5 Hz, 1H), 6.96 (td, *J* = 7.6, 1.5 Hz, 1H), 4.58 (d, *J* = 6.1 Hz, 2H), 3.70–3.62 (m, 4H), 3.19–3.10 (m, 4H), 2.35 (s, 3H). LCMS (Method 1) t_r_ = 6.93 min, *m*/*z* (M + H)^+^ = 471.1.

##### Compound **10**

^1^H NMR (400 MHz, DMSO-*d*_6_) δ 8.61 (t, *J* = 6.4 Hz, 1H), 7.45 (s, 1H), 7.22–7.11 (m, 3H), 7.10–6.91 (m, 5H), 4.37 (d, *J* = 6.4 Hz, 2H), 3.61–3.53 (m, 4H), 3.31–3.27 (m, 4H, overlaps with residual water peak), 2.26 (s, 3H). LCMS (Method 1) t_r_ = 6.28 min, *m*/*z* (M + H)^+^ = 471.0.

##### Compound **11**

^1^H NMR (400 MHz, DMSO-*d*_6_) δ 8.61 (t, *J* = 6.4 Hz, 1H), 8.15 (d, *J* = 2.7 Hz, 1H), 7.65 (dd, *J* = 9.1, 2.7 Hz, 1H), 7.46 (s, 1H), 7.20 (dd, *J* = 7.5, 7.5 Hz, 1H), 7.12–7.01 (m, 3H), 6.96 (d, *J* = 9.1 Hz, 1H), 4.39 (d, *J* = 6.4 Hz, 2H), 3.65 (dd, *J* = 6.8, 3.6 Hz, 4H), 3.57 (dd, *J* = 6.6, 3.6 Hz, 4H), 2.28 (s, 3H). LCMS (Method 1) t_r_ = 6.20 min, *m*/*z* (M + H)^+^ = 428.1.

##### Compound **12**

^1^H NMR (400 MHz, DMSO-*d*_6_) δ 8.61 (t, *J* = 6.5 Hz, 1H), 7.47 (s, 1H), 7.32–7.24 (m, 1H), 7.23–7.15 (m, 2H), 7.12–6.97 (m, 4H), 4.38 (d, *J* = 6.3 Hz, 2H), 3.63–3.56 (m, 4H), 3.30–3.23 (m, 5H), 2.28 (s, 3H). LCMS (Method 1) t_r_ = 6.21 min, *m*/*z* (M + H)^+^ = 445.1.

Compounds **13–20** were prepared according to the *General Procedure B*.

##### Compound **13**

^1^H NMR (400 MHz, DMSO-*d*_6_) δ 8.59 (t, *J* = 6.4 Hz, 1H), 7.54–7.40 (m, 2H), 7.30 (d, *J* = 7.5 Hz, 1H), 7.25 (s, 1H), 7.18 (t, *J* = 7.6 Hz, 1H), 7.12 (d, *J* = 7.6 Hz, 1H), 7.09–7.00 (m, 2H), 6.92 (d, *J* = 7.6 Hz, 1H), 4.38 (d, *J* = 6.3 Hz, 2H), 3.62 (t, *J* = 5.2 Hz, 4H), 3.39 (t, *J* = 5.3 Hz, 4H), 1.95–1.82 (m, 1H), 0.99–0.88 (m, 2H), 0.68–0.58 (m, 2H). LCMS (Method 1) t_r_ = 7.03 min, *m*/*z* (M + H)^+^ = 486.7.

##### Compound **14**

^1^H NMR (400 MHz, DMSO-*d*_6_) δ 8.71 (t, *J* = 6.4 Hz, 1H), 7.89 (d, *J* = 8.9 Hz, 2H), 7.79 (d, *J* = 7.7 Hz, 1H), 7.51–7.42 (m, 2H), 7.37 (t, *J* = 7.7 Hz, 1H), 7.33–7.21 (m, 3H), 7.12 (d, *J* = 7.6 Hz, 1H), 4.48 (d, *J* = 6.3 Hz, 2H), 3.64–3.60 (m, 4H, overlaps with residual water peak), 3.39 (t, *J* = 5.1 Hz, 4H), 2.71 (s, 3H). LCMS (Method 1) t_r_ = 6.77 min, *m*/*z* (M + H)^+^ = 543.7.

##### Compound **15**

^1^H NMR (400 MHz, DMSO-*d*_6_) δ 8.70 (t, *J* = 6.5 Hz, 1H), 7.53–7.40 (m, 2H), 7.35–7.23 (m, 4H), 7.17 (d, *J* = 7.7 Hz, 1H), 7.12 (d, *J* = 7.6 Hz, 1H), 4.38 (d, *J* = 6.3 Hz, 2H), 3.62 (t, *J* = 4.4 Hz, 4H), 3.39 (t, *J* = 5.2 Hz, 4H), 2.29 (s, 3H). LCMS (Method 1) t_r_ = 7.12 min, *m*/*z* (M + H)^+^ = 494.7.

##### Compound **16**

^1^H NMR (400 MHz, DMSO-*d*_6_) δ 8.68 (t, *J* = 6.4 Hz, 1H), 7.51–7.42 (m, 2H), 7.35 (d, *J* = 8.1 Hz, 1H), 7.33–7.23 (m, 3H), 7.17–7.09 (m, 2H), 4.38 (d, *J* = 6.3 Hz, 2H), 3.68–3.56 (m, 4H), 3.42–3.36 (m, 4H), 2.30 (s, 3H). LCMS (Method 1) t_r_ = 7.11 min, *m*/*z* (M + H)^+^ = 494.7.

##### Compound **17**

^1^H NMR (400 MHz, DMSO-*d*_6_) δ 8.76 (t, *J* = 6.4 Hz, 1H), 7.93–7.84 (m, 3H), 7.77 (s, 1H), 7.55–7.41 (m, 5H), 7.30 (d, *J* = 8.5 Hz, 1H), 7.25 (s, 1H), 7.12 (d, *J* = 7.6 Hz, 1H), 4.60 (d, *J* = 6.3 Hz, 2H), 3.69–3.58 (m, 4H), 3.42–3.36 (m, 4H). LCMS (Method 1) t_r_ = 7.01 min, *m*/*z* (M + H)^+^ = 496.7.

##### Compound **18**

^1^H NMR (400 MHz, DMSO-*d*_6_) δ 8.69 (t, *J* = 6.4 Hz, 1H), 7.52–7.42 (m, 2H), 7.41–7.23 (m, 8H), 7.12 (d, *J* = 7.6 Hz, 1H), 7.00 (d, *J* = 7.9 Hz, 2H), 6.93 (t, *J* = 7.3 Hz, 1H), 5.07 (s, 2H), 4.45 (d, *J* = 6.3 Hz, 2H), 3.62 (dd, *J* = 6.0, 4.1 Hz, 4H), 3.39 (dd, *J* = 6.7, 3.8 Hz, 4H). LCMS (Method 1) t_r_ = 7.08 min, *m*/*z* (M + H)^+^ = 553.2.

##### Compound **19**

^1^H NMR (400 MHz, DMSO-*d*_6_) δ 8.70 (t, *J* = 6.4 Hz, 1H), 7.57 (d, *J* = 2.1 Hz, 1H), 7.55–7.42 (m, 5H), 7.39 (dd, *J* = 7.6, 7.6 Hz, 1H), 7.33–7.22 (m, 5H), 7.12 (d, *J* = 7.6 Hz, 1H), 4.49 (d, *J* = 6.3 Hz, 2H), 3.67–3.57 (m, 4H), 3.41–3.35 (m, 4H), 2.33 (s, 3H). LCMS (Method 1) t_r_ = 7.40 min, *m*/*z* (M + H)^+^ = 536.7.

##### Compound **20**

^1^H NMR (400 MHz, DMSO-*d*_6_) δ 8.66 (t, *J* = 6.4 Hz, 1H), 7.50–7.42 (m, 2H), 7.35–7.27 (m, 3H), 7.25 (s, 1H), 7.19 (d, *J* = 8.2 Hz, 1H), 7.12 (d, *J* = 7.6 Hz, 1H), 7.02 (d, *J* = 7.6 Hz, 1H), 4.41 (d, *J* = 6.3 Hz, 2H), 3.68–3.57 (m, 6H), 3.41–3.38 (m, 4H, overlaps with residual water peak), 3.06 (t, *J* = 4.5 Hz, 2H). LCMS (Method 1) t_r_ = 6.13 min, *m*/*z* (M + H)^+^ = 516.2.

Synthesis of Compounds **21** (Supporting Information Figure S4).

Step 1. To a mixture of 2-(4-(3-(trifluoromethyl)­phenyl)­piperazin-1-yl)­thiazole-4-carboxylic acid (0.06 g, 0.168 mmol) and HATU (0.077 g, 0.201 mmol) in DMF (1.679 mL) under N_2_ atm was added DIPEA (0.044 mL, 0.252 mmol) and the mixture was stirred for 10 min. 3-(aminomethyl)­phenol (0.021 g, 0.168 mmol) was added to the above mixture followed by DIPEA (0.029 mL, 0.168 mmol) and the reaction mixture was left at stirring for 16 h. The desired product N-(3-hydroxybenzyl)-2-(4-(3-(trifluoromethyl)­phenyl)­piperazin-1-yl)­thiazole-4-carboxamide (0.054 g, 0.093 mmol) was purified by reverse phase flash column chromatography with MeCN (0.01% TFA) in H_2_O (0.01% TFA) 20 to 100% gradient.

Step 2. To a mixture of *N*-(3-hydroxybenzyl)-2-(4-(3-(trifluoromethyl)­phenyl)­piperazin-1-yl)­thiazole-4-carboxamide (0.0537 g, 0.116 mmol) and Cs_2_CO_3_ (0.076 g, 0.232 mmol) in DMF (0.774 mL) under N_2_ atm was added 3-(but-3-yn-1-yl)-3-(2-iodoethyl)-3*H*-diazirine (0.043 g, 0.174 mmol) and the mixture was stirred at 45 °C for 30 min. The desired compound was isolated by reverse phase flash column chromatography with MeCN (0.01% TFA) in H_2_O (0.01% TFA) 20 to 100% gradient. ^1^H NMR (400 MHz, Chloroform-*d*) δ 7.55 (t, *J* = 6.1 Hz, 1H), 7.50 (s, 1H), 7.39 (t, *J* = 8.1 Hz, 1H), 7.26 (d, *J* = 15.8 Hz, 2H), 7.18–7.13 (m, 2H), 7.10 (dd, *J* = 9.0, 2.3 Hz, 1H), 6.95 (d, *J* = 7.5 Hz, 1H), 6.89 (t, *J* = 2.2 Hz, 1H), 6.81 (dd, *J* = 8.2, 2.5 Hz, 1H), 4.59 (d, *J* = 6.1 Hz, 2H), 3.82 (t, *J* = 6.2 Hz, 2H), 3.69–3.60 (m, 4H), 3.40–3.29 (m, 4H), 2.06 (td, *J* = 7.5, 2.7 Hz, 2H), 1.98 (t, *J* = 2.6 Hz, 1H), 1.88 (t, *J* = 6.2 Hz, 2H), 1.73 (t, *J* = 7.5 Hz, 2H). ^13^C NMR (101 MHz, Chloroform-*d*) δ 170.6, 161.8, 158.9, 151.1, 146.1, 140.0, 131.8 (q, *J* = 31.9 Hz), 129.9, 124.3 (q, *J* = 272.6 Hz), 120.7, 119.8, 117.2 (q, *J* = 3.6 Hz), 114.2, 113.7,113.2(2) 113.2(0) (q, *J* = 3.8 Hz), 82.9, 69.4, 62.7, 48.6, 48.3, 43.4, 33.1, 32.8, 26.8, 13.4.

Purity of all final compounds was determined by LC-MS (or HPLC-MS) analysis and was confirmed to be ≥ 95%.

## Supplementary Material





## Data Availability

The computational workflows used in this study, including the DLCA scripts, Random Forest comparison scripts, and KNIME workflows for reaction enumeration, are publicly available in a GitHub repository: https://github.com/ncats/csar-reaction-enumeration.

## Notes

SMILES representations of the chemical structures for compounds listed in Tables S1–S5 (CSV)
ALDH3A1 biochemical and cellular assay data; selectivity and profiling data; benzaldehyde substrate-shift analysis; diazirine probe characterization and activity; photoaffinity-labeling proteomics and modeled binding pose; docking overlays; synthesis route to diazirine-containing probe, compound 21; and hit-to-lead efficiency metrics (Tables S1–S7 and Figures S1–S4) (PDF)

## Abbreviations Used

IC_50_: half-maximal activity concentration
ADME: absorption, distribution, metabolism, and excretion
AGC: automatic gain control
ALDH: aldehyde dehydrogenase
BAAA: BODIPY-aminoacetaldehyde
CSAR: comprehensive structure–activity relationship
CV: column volume
DEAB: diethylaminobenzaldehyde
DLCA: deep learning consensus architecture
DMSO: dimethyl sulfoxide
FA: formic acid
HCD: higher-energy collisional dissociation
HEPES: 4-(2-hydroxyethyl)-1-piperazineethanesulfonic acid
HPLC-MS/MS: high-performance liquid chromatography-tandem mass spectrometry
HTS: high-throughput screening
IAA: iodoacetamide
IC50: half-maximal inhibitory concentration
IMAC: immobilized metal affinity chromatography
IPTG: isopropyl β-d-1-thiogalactopyranoside
Km: Michaelis constant
KNIME: Konstanz Information Miner
LC-MS/MS: liquid chromatography–tandem mass spectrometry
MBP: maltose-binding protein
MOE: Molecular Operating Environment
MWCO: molecular weight cutoff
NAD­(P)+: nicotinamide adenine dinucleotide phosphate
OD600: optical density at 600 nm
PAMPA: parallel artificial membrane permeability assay
qHTS: quantitative high-throughput screening
QSAR: quantitative structure–activity relationship
RH: relative humidity
RT: room temperature
SAR: structure–activity relationship
SEC: size-exclusion chromatography
TCEP: tris­(2-carboxyethyl)­phosphine
TEV: tobacco etch virus
