# Supplementary material for: Integrating AI in Medicinal Chemistry for Accelerated Drug Discovery: A Comprehensive SAR (CSAR) Optimization Strategy and Discovery of Potent ALDH3A1 Inhibitors
Source: J Med Chem. 2026 May 30;69(11):13589–605. doi: 10.1021/acs.jmedchem.6c00537 (PMC13266984; doi:10.1021/acs.jmedchem.6c00537)
Supplement: Supplementary file 2 [file jm6c00537_si_002.pdf]

SUPPORTING INFORMATION

Integrating AI in Medicinal Chemistry for Accelerated Drug Discovery: A Comprehensive SAR (CSAR) Optimization Strategy and Discovery of Potent ALDH3A1 Inhibitors

Sankalp Jain[1], Adam Yasgar[1], Anu Dalal[1], Aleksandra Nilova[1], Marissa Davies[1], Bolormaa Baljinnyam[1], Yanyan Qu[1], John-Paul Denson[2], Dominic Esposito[2], Dingyin Tao[1], Shyh-Ming Yang[1], Daniel C. Talley[1], Anton Simeonov[1], Natalia J. Martinez[1], Ganesha Rai[1]*, Alexey V. Zakharov[1]*

[1]National Center for Advancing Translational Sciences (NCATS), National Institutes of Health, 9800 Medical Center Drive, Rockville, MD, 20850, United States

[2]Protein Expression Laboratory, Cancer Research Technology Program, Frederick National Laboratory for Cancer Research, Frederick, MD, 21701, United States

*Address for correspondence: 9800 Medical Center Dr, Rockville, Maryland 20850, USA; Telephone: 301-480-9847; E-mails: bantukallug@mail.nih.gov and alexey.zakharov@nih.gov.

Table of Contents

Table S1. ALDH3A1 biochemical activity for compounds tested in the primary screen S3
Table S2. ALDH3A1 biochemical activity for analogs tested S4
Table S3. RF Phase I QSAR model performance across alternative molecular representations S7
Table S4. CSAR Phase I reaction-based enumeration at R_1: synthesized analogs and activity data S7
Table S5. CSAR Phase II reaction-based enumeration at R_2: synthesized analogs and activity data S9
Figure S1. Effect of increased benzaldehyde concentration on inhibition by NCATS-SM0707 and NCATS-SM0708 .... S13
Table S6. Effect of increased benzaldehyde concentration on biochemical potency S13
Figure S2. Diazirine probe characterization, activity, proteomics, and modeled binding pose S14
Table S7. Hit-to-lead optimization metrics for the initial hit and optimized ALDH3A1 inhibitors S15
Figure S3. Docking-based comparison of the original hit and NCATS-SM0707 .... S16
Figure S4. Synthesis route to diazirine-containing probe, compound 21 .... S17

SMILES representations of the chemical structures for compounds listed in Tables S1–S5 are provided in the accompanying compound_smiles.csv.xlsx file.

Table S1. ALDH3A1 biochemical activity for compounds tested in the primary screen, including compound identifiers and potency readouts.

Sample ID	Compound ID (manuscript)	AC50 (uM)	CC-v2	Efficacy
NCGC00531207-01		14.13	-2.3	-93.27
NCGC00433986-01		14.13	-2.2	-81.82
NCGC00422389-01		15.85	-3	-81.78
NCGC00394846-01		15.85	-2.2	-70.18
NCGC00427669-01		3.98	-1.2	-69.95
NCGC00435271-01		19.95	-3	-66.49
NCGC00398253-01		14.13	-2.2	-63.09
NCGC00439852-01		0.89	-1.2	-61.76
NCGC00528999-01		14.13	-2.4	-59.29
NCGC00445370-01		14.13	-2.2	-58.59
NCGC00426725-01		28.18	-2.2	-57.51
NCGC00530028-01		19.95	-2.2	-57.15
NCGC00518899-01		12.59	-2.2	-52.83
NCGC00473302-01		28.18	-2.4	-52.44

NCGC00527311-01		28.18	-2.2	-52.11
NCGC00458073-01		39.81	-3	-51.44
NCGC00411020-01		14.13	-2.2	-49.01
NCGC00441061-01		31.62	-2.2	-48.80
NCGC00527314-01		35.48	-2.4	-47.14
NCGC00449252-01		15.85	-2.2	-45.94
NCGC00396353-01		35.48	-2.2	-44.61
NCGC00526793-01		35.48	-2.2	-44.11
NCGC00475032-01		15.85	-2.4	-43.09
NCGC00416184-01		35.48	-2.4	-42.40
NCGC00438506-01		5.62	-2.4	-41.70
NCGC00438461-01		35.48	-2.4	-41.61
NCGC00393854-01		31.62	-2.2	-39.60
NCGC00428876-01		31.62	-2.2	-39.16
NCGC00400077-01		39.81	-2.2	-39.06
NCGC00412778-01		44.67	-2.2	-38.45

NCGC00429361-01		28.18	-3	-36.95
NCGC00433095-01		28.18	-2.4	-36.91
NCGC00426329-01		15.85	-3	-34.89
NCGC00529385-01		11.22	-2.2	-31.52
NCGC00449346-01		4.47	-1.2	-31.03
NCGC00511907-01		17.78	-2.2	-30.84
NCGC00398388-01		28.18	-2.4	-30.64
NCGC00501442-01		8.91	-1.4	-29.51
NCGC00438182-01		2.51	-1.4	-29.34
NCGC00373680-01		7.94	-1.4	-29.03
NCGC00437746-01		28.18	-2.4	-25.70
NCGC00441819-01		15.85	-2.4	-25.02
NCGC00401096-01		8.91	-1.4	-24.34
NCGC00396390-01		56.23	-2.4	-23.02
NCGC00450299-01		10.00	-1.4	-21.09
NCGC00398210-01		22.39	-2.4	-20.43

NCGC00398197-01		35.48	-2.4	-17.95

Table S2. ALDH3A1 biochemical activity for analogs tested, including compound identifiers and potency readouts.

Sample ID	Compound ID (manuscript)	AC50 (uM)	CC-v2	Efficacy
NCGC00430682-01	2	2.51	-1.2	-72.54
NCGC00430668-01		3.98	-1.2	-57.20
NCGC00417081-01		15.85	-2.2	-39.10
NCGC00430728-01		28.18	-2.4	-37.07
NCGC00430720-01		15.85	-2.2	-57.30
NCGC00392637-01		12.59	-2.2	-37.26
NCGC00392964-01		0.32	-1.2	-32.94
NCGC00392993-01		0.32	-1.4	-23.33
NCGC00430672-01		0.11	-1.2	-32.51
NCGC00421409-01		10.00	-1.2	-39.09
NCGC00394621-01		3.98	-1.1	-97.74
NCGC00430666-01		3.55	-1.2	-54.85
NCGC00399959-01		28.18	-2.2	-62.15

NCGC00391691-01		22.39	-2.2	-46.23
NCGC00417077-01		28.18	-2.2	-48.69
NCGC00391544-01		25.12	-2.4	-31.39
NCGC00430692-01	1	0.71	-1.2	-61.67
NCGC00430648-01		25.12	-2.2	-56.53
NCGC00430686-01		12.59	-2.2	-75.42
NCGC00391075-01		15.85	-2.4	-69.78
NCGC00412294-01		12.59	-1.1	-83.58
NCGC00392991-01		4.47	-2.4	-26.14
NCGC00417051-01		3.16	-1.2	-53.13
NCGC00412345-01		5.62	-2.4	-73.33
NCGC00430670-01		0.25	-1.4	-27.44
NCGC00412252-01		0.25	-1.4	-26.81
NCGC00430690-01	3	12.59	-2.2	-54.71
NCGC00430664-01		4.47	-1.4	-32.83
NCGC00401594-01		12.59	-2.2	-91.30
NCGC00417023-01		6.31	-2.2	-62.10

NCGC00430738-01		1.41	-1.1	-92.95
NCGC00430642-01		1.41	-1.2	-74.85
NCGC00430730-01		6.31	-1.2	-78.52
NCGC00392980-01		0.18	-1.4	-21.56
NCGC00417099-01		0.79	-1.4	-54.49
NCGC00430684-01		14.13	-2.2	-68.54
NCGC00430718-01		2.51	-1.2	-65.44
NCGC00417124-01		2.82	-2.2	-61.06
NCGC00430676-01		1.00	-1.2	-50.47
NCGC00430704-01		15.85	-2.4	-42.05
NCGC00394633-01		15.85	-2.2	-62.62
NCGC00392526-01		28.18	-3	-67.89
NCGC00417061-01		0.63	-1.2	-48.22
NCGC00397524-03		3.16	-1.2	-51.19
NCGC00417155-01		5.62	-1.2	-82.24
NCGC00430734-01		1.78	-1.2	-71.11
NCGC00391090-01		15.85	-1.2	-53.10

NCGC00430688-01		2.82	-1.2	-45.69
NCGC00399960-01		14.13	-1.1	-93.02
NCGC00395823-01		14.13	-2.1	-101.87
NCGC00400191-01		22.39	-2.2	-83.17
NCGC00397796-01		14.13	-2.1	-119.47
NCGC00417040-01		19.95	-2.4	-27.04
NCGC00417031-01		14.13	-2.2	-80.78
NCGC00430726-01		2.00	-1.2	-64.54
NCGC00391159-01		0.79	-1.1	-109.85
NCGC00417071-01		15.85	-2.2	-40.37
NCGC00417085-01		28.18	-2.2	-77.64
NCGC00430698-01		2.00	-1.2	-75.40
NCGC00393065-01		0.45	-1.1	-92.90
NCGC00394616-01		28.18	-2.1	-105.83
NCGC00417015-01		17.78	-2.2	-40.16
NCGC00417044-01		17.78	-2.2	-40.76
NCGC00392986-01		28.18	-2.2	-62.94

NCGC00430736-01		7.08	-1.2	-65.35

Table S3. RF Phase I QSAR model performance across alternative molecular representations.

	Average R^2 (5-fold CV)	Average RMSE (5-fold
RDKit descriptors	0.1474	0.5172
Morgan fingerprint	0.1766	0.5083
Avalon fingerprint	0.1415	0.519
AtomPair fingerprint	0.1456	0.5178
Combined feature	0.1822	0.5066

Table S4. CSAR Phase I (reaction-based enumeration at R_1): synthesis of prioritized analogs, including compound identifiers and ALDH3A1 biochemical and cellular activity readouts.

Sample ID	Compound ID (manuscript)	AC50 (uM)	CC-v2	Efficacy	Cell_AC50 (uM)	Cell_CC-v2	Cell_Efficacy
NCGC01219263-01	5	0.23	-1.1	-100.07	3.13	-2.2	-101.21
NCGC01211118-01	6	0.32	-1.1	-79.10	1.97	-1.1	-102.70
NCGC01211130-01	7	0.32	-1.1	-97.93	0.88	-1.1	-93.28
NCGC01211137-01	8	0.36	-1.1	-84.72	0.50	-2.1	-115.29
NCGC01211373-01		0.36	-1.2	-60.22	1.11	-1.1	-85.56
NCGC01211192-01	9	0.41	-1.1	-99.96	8.81	-1.3	-81.02
NCGC01212473-01	10	0.41	-1.1	-94.28	0.20	-1.3	-86.93
NCGC01211114-01		0.46	-1.2	-48.74	1.57	-1.1	-79.67

NCGC01212471-01		0.46	-1.1	-100.78	?	4	-20.41
NCGC01219352-01	11	0.46	-1.1	-99.36	3.51	-1.2	-68.27
NCGC01211131-01		0.51	-1.1	-89.06	3.13	-1.4	-61.19
NCGC01211125-01	12	0.51	-1.1	-99.84	0.99	-1.1	-76.59
NCGC01211120-01		0.51	-1.1	-83.25	0.79	-1.1	-88.59
NCGC01210241-01		0.58	-1.1	-95.65	22.13	-2.4	-83.77
NCGC01210535-01		0.58	-1.1	-96.11	7.85	-1.1	-90.86
NCGC01211545-01		0.58	-1.2	-72.19	0.39	-1.2	-73.43
NCGC01211398-01		0.58	-1.1	-91.75	2.48	-1.1	-99.68
NCGC01211381-01		0.58	-1.1	-89.43	31.26	-2.3	-102.69
NCGC01211123-01		0.58	-1.2	-77.80	1.76	-1.2	-94.45
NCGC01211133-01		0.65	-1.1	-87.41	1.57	-1.1	-95.95
NCGC01211188-01		0.65	-1.2	-69.38	1.76	-1.1	-81.68
NCGC01211384-01		0.65	-1.1	-96.17	3.93	-1.1	-82.59
NCGC01210237-01		0.73	-1.1	-94.33	3.13	-1.3	-106.55
NCGC01210611-01		0.73	-1.2	-77.35	0.62	-1.2	-33.71
NCGC01219289-01		0.73	-1.1	-97.63	0.99	-1.1	-93.51

NCGC01210239-01		0.81	-1.1	-79.44	9.88	-2.4	-51.72
NCGC01211165-01		0.81	-1.2	-72.77	0.70	-1.1	-91.24
NCGC01210996-01		0.91	-1.1	-91.28	0.05	-1.3	-98.32
NCGC01211383-01		0.91	-1.1	-88.99	15.67	-2.1	-112.70
NCGC01211189-01		0.91	-1.1	-85.97	7.85	-1.1	-96.01
NCGC01211187-01		0.91	-1.1	-93.83	4.42	-2.1	-121.01
NCGC01211121-01		0.91	-1.1	-88.53	12.44	-1.3	-81.66
NCGC01211128-01		0.91	-1.1	-85.15	3.13	-1.1	-86.59
NCGC01210654-01		1.02	-1.1	-95.69	35.07	-2.2	-81.56
NCGC01210664-01		1.02	-1.1	-83.70	11.09	-2.1	-103.93
NCGC01210598-01		1.02	-1.2	-62.83	3.93	-2.1	-104.17
NCGC01211295-01		1.02	-1.1	-92.09	2.21	-1.3	-85.13
NCGC01211127-01		1.02	-1.1	-98.80	4.95	-1.1	-89.89
NCGC01219288-01		1.02	-1.1	-96.39	0.99	-1.1	-85.92
NCGC01211174-01		1.15	-1.2	-66.83	8.81	-1.1	-87.39
NCGC01211394-01		1.15	-1.2	-71.12	15.67	-2.3	-88.85
NCGC01211399-01		1.15	-1.2	-77.88	5.56	-1.3	-101.59

NCGC01211171-01		1.15	-1.2	-53.27	1.97	-1.1	-83.98
NCGC01219292-01		1.15	-1.1	-94.91	0.88	-1.3	-90.33
NCGC01211115-01		1.29	-1.2	-72.39	0.99	-1.1	-81.24
NCGC01211588-01		1.29	-1.2	-45.52	1.97	-1.1	-97.51
NCGC01212812-01		1.29	-1.1	-84.38	0.79	-1.1	-81.50
NCGC01212814-01		1.29	-1.1	-92.59	?	4	0.00
NCGC01211138-01		1.45	-1.1	-88.86	7.85	-1.1	-88.54
NCGC01211190-01		1.45	-1.2	-61.92	3.51	-1.1	-84.69
NCGC01211169-01		2.05	-1.1	-94.63	8.81	-1.1	-88.08
NCGC01211117-01		2.05	-1.1	-84.99	8.81	-1.1	-97.52
NCGC01211119-01		2.57	-1.2	-61.43	15.67	-1.2	-77.14
NCGC01211377-01		2.57	-1.1	-107.89	12.44	-2.3	-107.62
NCGC01211397-01		2.89	-1.2	-71.38	2.79	-1.1	-82.14
NCGC01211173-01		3.24	-1.2	-45.43	1.40	-1.1	-87.33
NCGC01211124-01		3.24	-1.2	-88.96	11.09	-2.2	-100.20
NCGC01212809-01		3.24	-1.1	-99.09	3.93	-1.1	-80.26
NCGC01210723-01		3.64	-1.2	-72.64	1.24	-1.2	-75.10

NCGC01211386-01		3.64	-1.2	-76.22	9.88	-1.2	-70.97
NCGC01211122-01		4.08	-1.2	-67.64	7.85	-1.1	-90.75
NCGC01211186-01		4.08	-1.2	-37.13	3.13	-1.1	-98.82
NCGC01211126-01		5.76	-1.2	-62.10	15.67	-1.2	-68.34
NCGC01211172-01		5.76	-1.1	-86.01	8.81	-1.1	-92.43
NCGC01211669-01		6.47	-1.2	-51.05	8.81	-1.1	-91.42
NCGC01211296-01		10.25	-1.2	-82.31	13.96	-1.1	-111.44
NCGC01211191-01		10.25	-1.2	-70.10	9.88	-1.1	-90.32
NCGC01210256-01		14.48	-1.2	-66.81	15.67	-1.4	-71.00
NCGC01211132-01		14.48	-2.1	-105.48	13.96	-2.4	-92.53
NCGC01210726-01		18.23	-1.2	-54.32	15.67	-1.2	-81.37
NCGC01211194-01		40.80	-2.2	-38.00	0.88	-1.2	-43.91
NCGC01211184-01		?	4	-26.97	0.31	-1.1	-95.63

Table S5. CSAR Phase II (reaction-based enumeration at R2): synthesis of prioritized analogs, including compound identifiers and ALDH3A1 biochemical and cellular activity readouts.

Sample ID	Compound ID (manuscript)	NCATS Name	AC50 (uM)	CC-v2	Efficacy	Cell_AC50 (uM)	Cell_CC-v2	Cell_Efficacy
NCGC01407980-01			15.8489	-1.1	-102.93	0.0447	-1.2	-75.54

NCGC01408046-01			15.8489	-1.1	-91.30	?	4	-23.84
NCGC01407817-01			8.9125	-1.2	-57.06	0.0316	-1.1	-111.10
NCGC01407976-01			2.5119	-1.2	-33.77	?	5	0.00
NCGC01397384-01			1.5849	-1.2	-41.09	?	4	-16.71
NCGC01397296-01			1.1220	-1.2	-67.67	35.4813	-2.3	-82.41
NCGC01407971-01			0.8913	-1.2	-53.81	0.2512	-1.1	-83.43
NCGC01408044-01			0.8913	-1.2	-74.78	0.0112	5	-68.37
NCGC01407779-01			0.7943	-1.2	-73.90	2.2387	-1.1	-130.58
NCGC01397276-01			0.7943	-1.2	-44.50	0.0112	5	-27.70
NCGC01397385-01			0.7079	-1.2	-71.46	17.7828	5	23.55
NCGC01407999-01			0.7079	-1.2	-44.75	0.0079	-1.3	-93.07
NCGC01407808-01			0.6310	-1.4	-20.90	0.2239	-1.3	-75.55
NCGC01407948-01			0.6310	-1.2	-52.77	?	5	0.00
NCGC01407986-01			0.5623	-1.2	-31.39	0.5012	-1.1	-92.16
NCGC01407763-01			0.5623	5	-16.45	?	4	0.00
NCGC01407819-01			0.5012	-1.1	-83.40	0.3162	-1.1	-103.28
NCGC01407797-01			0.5012	5	-19.41	?	4	-19.71
NCGC01397286-01			0.4467	-1.2	-60.58	0.3981	5	-10.99
NCGC01407765-01			0.4467	-1.2	-37.12	0.2818	-1.2	-76.28
NCGC01407818-01			0.4467	-1.2	-62.88	0.1995	-1.1	-88.67
NCGC01397299-01			0.4467	-1.2	-37.47	0.0794	-1.2	-61.99
NCGC01407770-01			0.4467	-1.2	-78.43	0.0794	-1.1	-87.64
NCGC01407922-01			0.3981	-1.4	-26.41	2.2387	-1.1	-108.81
NCGC01407783-01			0.3981	-1.1	-80.39	0.0316	-1.3	-92.90
NCGC01397290-02			0.3981	-1.2	-53.79	?	5	0.00
NCGC01407759-01			0.3981	-1.4	-29.26	?	5	0.00
NCGC01407975-01			0.3548	-1.2	-39.06	0.1000	-1.1	-85.75
NCGC01407768-01			0.3162	-1.2	-32.49	15.8489	-1.3	-93.67
NCGC01407816-01			0.1995	5	-21.51	19.9526	-1.1	-99.22

NCGC01407706-01			0.0892	-1.2	-65.00	?	4	0.00
NCGC01407857-01			0.0795	-1.2	-80.62	0.0193	-1.1	-77.57
NCGC01397294-01			0.0782	-1.2	-73.44	8.4628	-1.3	-106.03
NCGC01407985-01			0.0750	-1.2	-78.48	0.0910	-1.1	-83.99
NCGC01407937-01	20		0.0631	-1.1	-96.26	0.0108	-1.2	-81.94
NCGC01407979-01			0.0631	-1.1	-94.05	0.3423	-1.3	-96.60
NCGC01407772-01			0.0596	-1.1	-88.60	0.6444	-1.1	-100.81
NCGC01407972-01			0.0501	-1.1	-90.79	?	4	0.00
NCGC01396481-01			0.0398	-1.1	-98.89	0.3423	-1.4	-73.69
NCGC01407951-01			0.0355	-1.1	-100.75	4.3096	-1.3	-93.55
NCGC01396421-01	14	NCATS-SM0708	0.0316	-1.1	-99.22	0.0038	-1.1	-94.02
NCGC01407933-01			0.0282	-1.1	-87.34	2.7192	-1.1	-85.12
NCGC01407914-01			0.0282	-1.1	-95.28	0.6087	-1.3	-99.51
NCGC01407934-01			0.0282	-1.1	-101.06	0.3423	-1.1	-95.79
NCGC01407771-01			0.0251	-1.1	-94.50	3.0510	-1.2	-89.96
NCGC01397390-01			0.0251	-1.1	-96.99	1.9250	-1.1	-76.04
NCGC01397289-01			0.0251	-1.1	-100.97	0.3423	-1.3	-79.11
NCGC01407982-01			0.0237	-1.1	-97.73	0.8113	-1.2	-88.39
NCGC01407760-01	19		0.0237	-1.1	-83.86	0.0102	-1.1	-77.01
NCGC01397292-01			0.0200	-1.1	-100.00	0.6830	-2.1	-111.50
NCGC01407784-01			0.0188	-1.1	-99.55	0.0407	-1.2	-74.47
NCGC01407978-01			0.0178	-1.1	-100.27	1.5291	-2.1	-119.85
NCGC01407942-01			0.0178	-1.1	-100.07	?	4	0.00
NCGC01396486-01			0.0159	-1.1	-100.89	?	4	0.00
NCGC01407809-01			0.0141	-1.1	-101.87	9.6480	-1.4	-60.41
NCGC01407935-01			0.0141	-1.1	-100.75	1.0825	-1.1	-91.56
NCGC01407984-01			0.0141	-1.1	-99.03	0.2423	-2.2	-105.03
NCGC01407943-01	18		0.0141	-1.1	-100.33	0.0431	-1.1	-79.42
NCGC01407936-01			0.0126	-1.1	-100.15	0.9648	-1.1	-88.16

NCGC01407815-01			0.0126	-1.1	-94.32	?	4	38.41
NCGC01407769-01	17		0.0119	-1.1	-92.05	0.0407	-1.1	-93.35
NCGC01396480-01			0.0112	-1.1	-96.06	1.3628	-1.4	-77.80
NCGC01407930-01			0.0112	-1.1	-103.16	0.8599	-2.1	-116.21
NCGC01407812-01			0.0100	-1.1	-101.90	1.2146	-1.2	-65.48
NCGC01407701-01	16		0.0079	-1.1	-106.26	0.0342	-1.1	-81.01
NCGC01397293-01			0.0056	-1.1	-100.05	0.7664	-1.1	-101.81
NCGC01397331-01			0.0040	-1.1	-97.11	3.0510	-1.3	-82.49
NCGC01397297-01			0.0036	-1.1	-107.41	2.1599	-1.1	-85.43
NCGC01407805-01			0.0028	-1.1	-102.00	0.9648	-1.4	-69.34
NCGC01397393-01			0.0022	-1.1	-100.26	0.9648	-2.2	-104.84
NCGC01397391-01	15		0.0020	-1.1	-106.28	0.0860	-1.1	-77.59
NCGC01397386-01	13	NCATS-SM0707	0.0011	-1.1	-107.26	0.0136	-1.2	-65.41
NCGC01407977-01			?	4	-16.69	50.1187	5	-33.22
NCGC01407961-01			?	4	-23.04	28.1838	-2.3	-38.73
NCGC01407762-01			?	4	0.00	11.2202	-1.4	-62.69
NCGC01397389-01			?	4	-20.50	7.0795	-2.1	-125.53
NCGC01396478-01			?	4	-22.34	6.3096	-2.1	-114.29
NCGC01407780-01			?	4	-7.18	?	4	0.00

A) B)

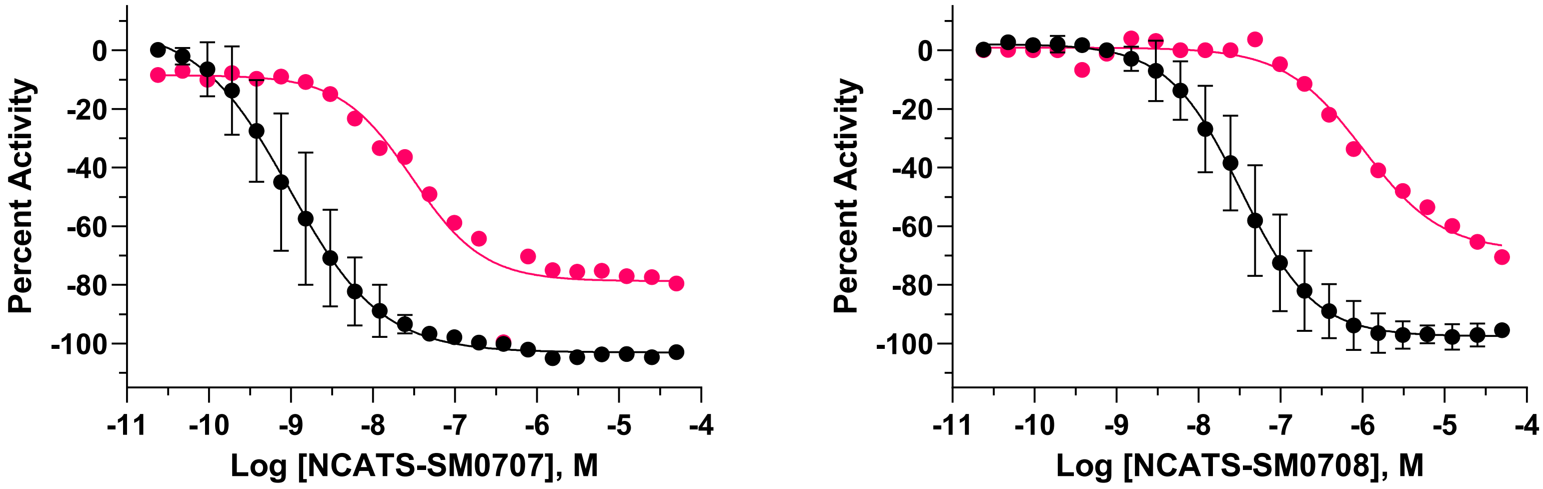

Figure S1. Effect of increased benzaldehyde concentration on inhibition by NCATS-SM0707 and NCATS-SM0708. Biochemical dose-response curves for NCATS-SM0707 (A) and NCATS-SM0708 (B) measured under the standard assay condition using benzaldehyde at 200 µM (•) and 4,000 µM (•), corresponding to an approximately 20-fold increase in K_m. In both cases, increasing benzaldehyde produced a pronounced rightward shift in the concentration-response curve, consistent with substrate-competitive inhibition. The cofactor, $NADP^+$ was maintained at 1,000 µM (~4× Km) as a bias against competition with the cofactor.

Table S6. Effect of increased benzaldehyde concentration on the biochemical potency of NCATS-SM0707 and NCATS-SM0708.

**Sample ID (NCATS)**	**20X[SUB] IC_{50} [µM]**	**1X[SUB] IC_{50} [µM]**	**IC_{50}Ratio**
NCATS-SM0707	0.0316	0.00112	28

NCATS-SM0708	1	0.0316	32

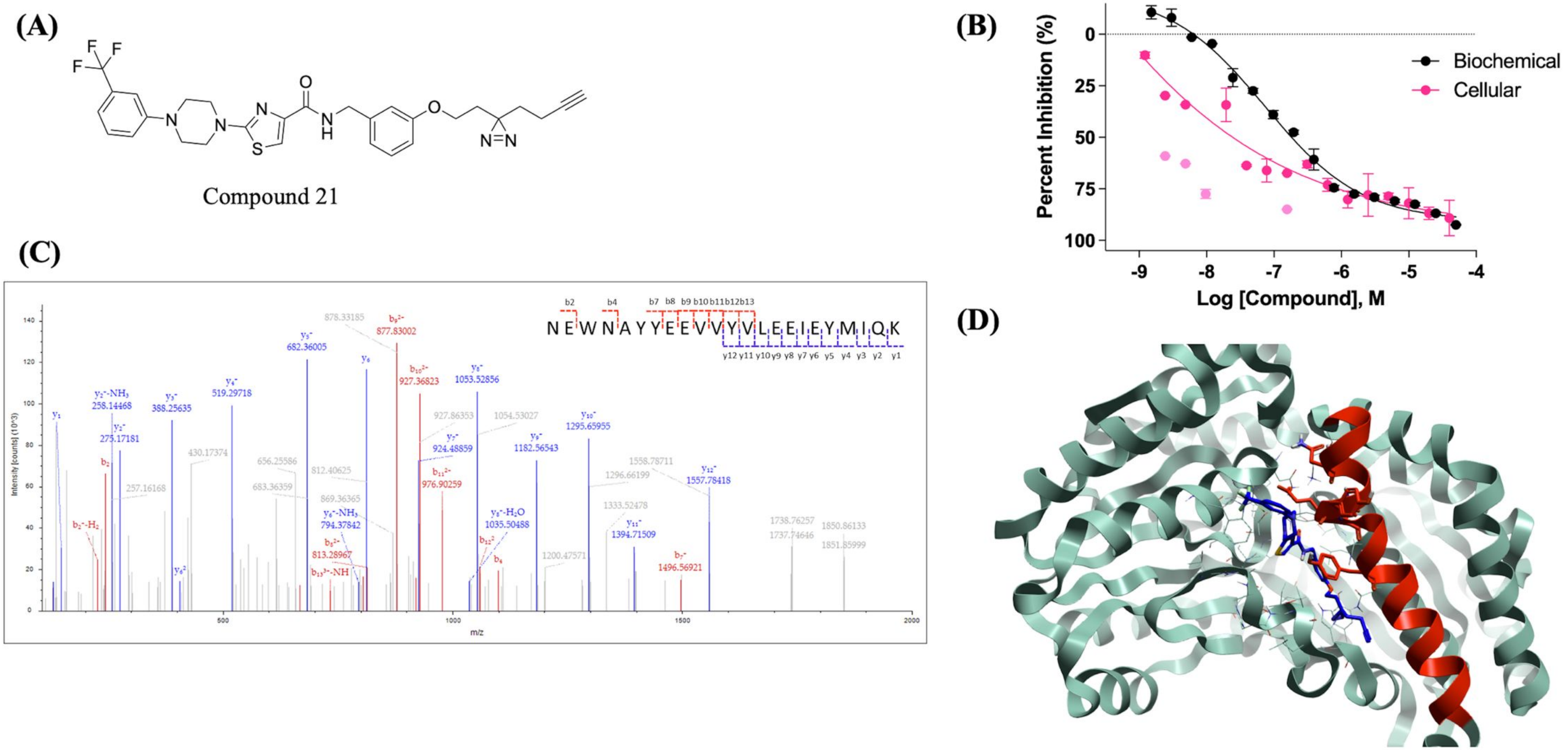

Figure S2. Diazirine probe characterization, activity, proteomics, and modeled binding pose.

(A) Molecular structure of diazirine derivative of compound **13 (NCATS-SM0707)**

(B) Activity of compound **21** in ALDH3A1 biochemical and cellular assays

(C) Peptide sequence identified to interact with compound **21** and its MS/MS spectra

(D) Modeled binding pose of compound **21** and compound **13 (NCATS-SM0707)** with diazirine in ALDH3A1. ALDH3A1 is shown as a teal ribbon, with compound **21** (diazirine probe) in blue sticks and mass-spectrometry–identified modified residues highlighted in red on the α-helical segment lining the active site.

Table S7. Hit-to-lead optimization metrics for the initial hit and optimized ALDH3A1 inhibitors.

Sample ID	Name	AC50_uM	MW	HeavyAtomCount	cLogP	pAC50	LE	LLE
NCGC00430642-01	initial hit	1.41	392.53	28	3.71	5.851	0.286	2.143
NCGC01397386-01	NCATS-SM0707	0.0011	486.56	34	5.3	8.959	0.361	3.663
NCGC01396421-01	NCATS-SM0708	0.0316	543.64	37	5.85	7.5	0.278	1.65

pAC50 = 6 − log10(AC50 in μM)
LE = 1.37 × pAC50 / HeavyAtomCount
LLE = pAC50 − cLogP

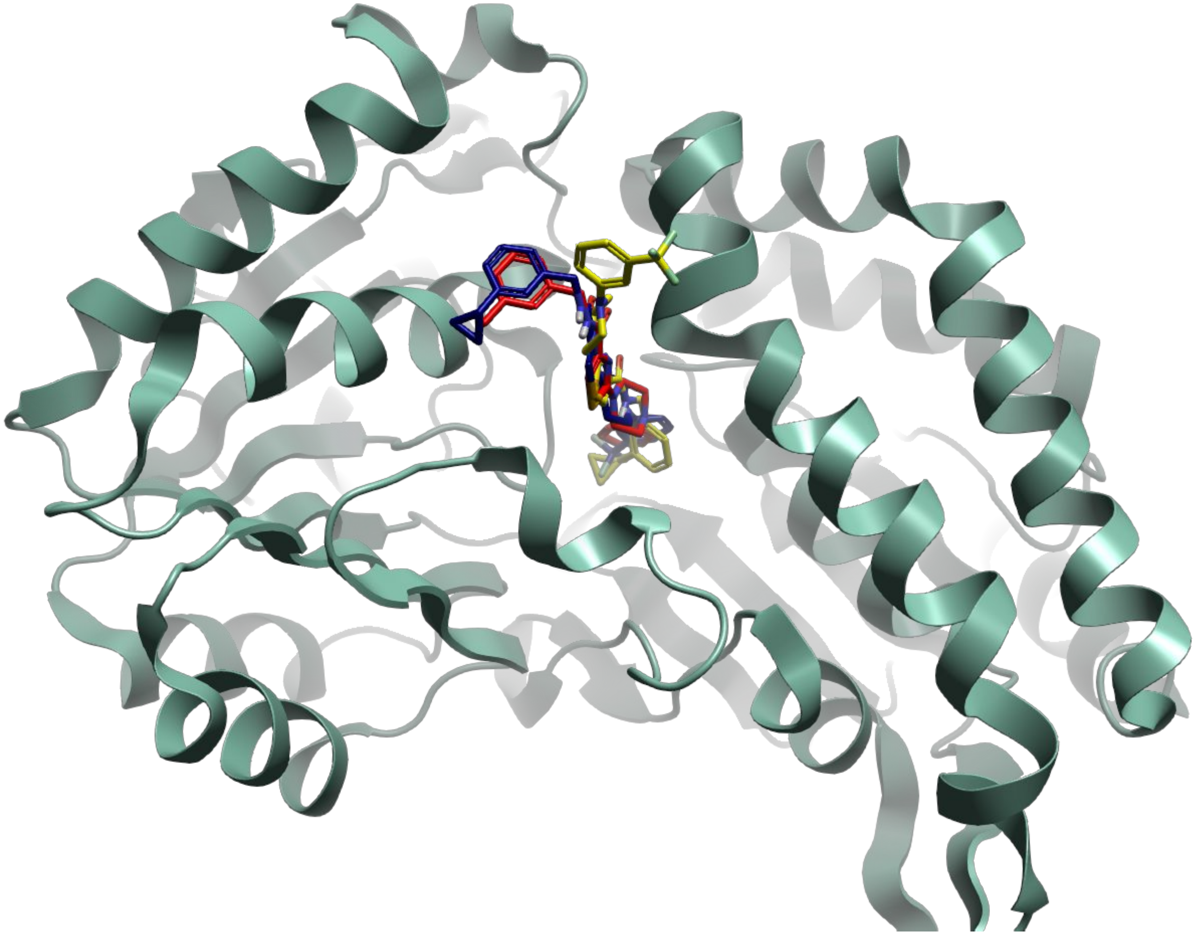

Figure S3. Docking-based comparison of the original hit and NCATS-SM0707 in the ALDH3A1 binding pocket. The docked pose of the original hit is shown in red. Two representative poses of NCATS-SM0707 are shown, including one aligned with the original hit pose (blue) and one flipped orientation (yellow). These poses indicate that NCATS-SM0707 remains compatible with the same general substrate-binding region as the original scaffold, although the precise binding geometry cannot be assigned unambiguously from docking alone. The docking results support preserved pocket engagement and improved overall complementarity during optimization, in agreement with the photoaffinity-labeling data.

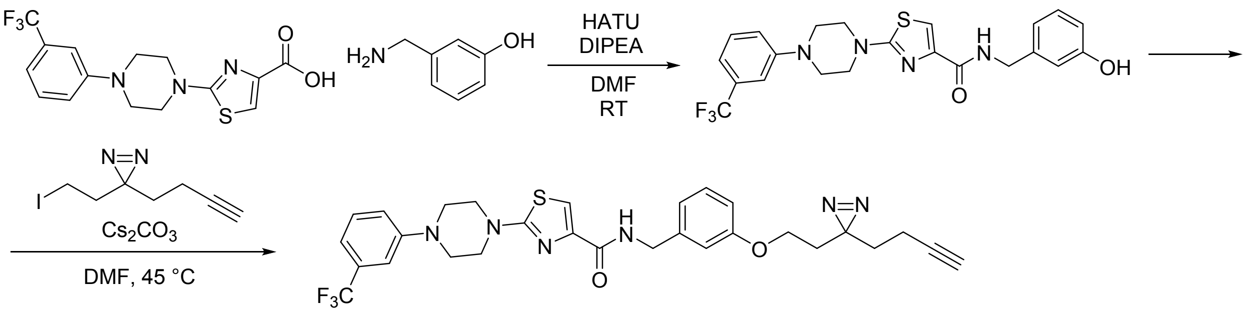

Figure S4. Synthesis route to diazirine-containing probe, compound 21.
